# ﻿Nine new spider species of *Belisana* Thorell, 1898 (Araneae, Pholcidae) from karst caves, with a list of species of the genus from Guizhou, southwestern China

**DOI:** 10.3897/zookeys.1216.132561

**Published:** 2024-10-25

**Authors:** Bing Wang, Jinglin Li, Shuqiang Li, Zhiyuan Yao

**Affiliations:** 1 College of Life Science, Shenyang Normal University, Shenyang 110034, Liaoning, China Shenyang Normal University Shenyang China; 2 Institute of Zoology, Chinese Academy of Sciences, Beijing 100101, China Institute of Zoology, Chinese Academy of sciences Beijing China

**Keywords:** Biodiversity, cellar spiders, daddy-long-legs, invertebrate, morphology, taxonomy

## Abstract

Species of the spider genus *Belisana* Thorell, 1898 exhibit high diversity in Guizhou, southwestern China. Previously, only eight species of *Belisana* were recorded in Guizhou. In this study, nine new species are described from karst caves: *Belisanabijie* Wang, S. Li & Yao, **sp. nov.**, *B.liupanshui* Wang, S. Li & Yao, **sp. nov.**, *B.majiang* Wang, S. Li & Yao, **sp. nov.**, *B.nayong* Wang, S. Li & Yao, **sp. nov.**, *B.qixingguan* Wang, S. Li & Yao, **sp. nov.**, *B.xiuwen* Wang, S. Li & Yao, **sp. nov.**, *B.yongcong* Wang, S. Li & Yao, **sp. nov.**, *B.zhouxi* Wang, S. Li & Yao, **sp. nov.**, and *B.zunyi* Wang, S. Li & Yao, **sp. nov.***Belisanazhangi* Tong & Li, 2007 is reported from Guizhou for the first time. In addition, a list of all species of *Belisana* from Guizhou is provided.

## ﻿Introduction

*Belisana* Thorell, 1898, the second largest genus in the Pholcidae C.L. Koch, 1850, comprises 160 species (WSC 2024). These species inhabit diverse micro-habitats, e.g., beneath rocks, in caves, on the underside of leaves, among leaf litter, and amidst foliage in the canopy (Huber 2005; Yao et al. 2015; Zhao et al. 2023a). They are primarily distributed in southern China, as well as in the Indo-Malayan and Australasian regions (Huber 2005; Yao and Li 2013; Yao et al. 2018; Zhu et al. 2020; Zhu and Li 2021). Currently, 46% of the species (74 spp.) have been documented from southern China (WSC 2024), of which Yunnan boasts the highest concentration of species, accounting for 42% (31 spp.; Zhang et al. 2024b). Guangxi and Hainan, which have the second and third highest species diversity of *Belisana*, respectively, have recorded only 11 and ten species. Recently, numerous surveys targeting pholcid spiders have been conducted in China, resulting in the discovery and reporting of a large number of new species (e.g., Yao et al. 2021; Lu et al. 2022; Zhao et al. 2023b; Yang et al. 2024a, b). Nevertheless, these efforts have primarily focused on *Pholcus* Walckenaer, 1805, found in epigean environments in northern and central China, with relatively few reports on *Belisana* from hypogean environments in southern China (10 spp.; Wang et al. 2024; Zhang et al. 2024a, b).

**Figure 1.**
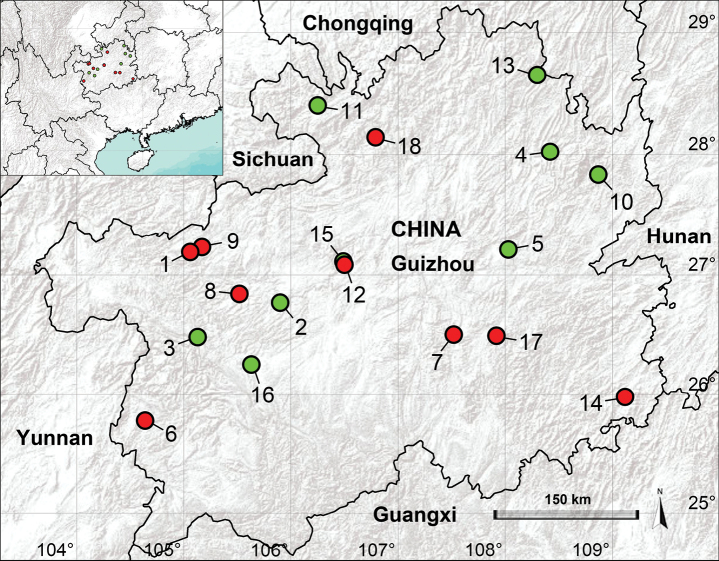
Distribution records of the *Belisana* species from Guizhou, China **1***Belisanabijie* sp. nov. **2***B.daji* Chen, Zhang & Zhu, 2009 **3***B.douqing* Chen, Zhang & Zhu, 2009 **4***B.galeiformis* Zhang & Peng, 2011 **5***B.lii* Chen, Yu & Guo, 2016 **6***B.liupanshui* sp. nov. **7***B.majiang* sp. nov. **8***B.nayong* sp. nov. **9***B.qixingguan* sp. nov. **10***B.wangchengi* Wang, Yao & Zhang, 2024 **11***B.xishui* Chen, Zhang & Zhu, 2009 **12***B.xiuwen* sp. nov. **13***B.yanhe* Chen, Zhang & Zhu, 2009 **14***B.yongcong* sp. nov. **15***B.yuhaoi* Yang & Yao, 2023 **16***B.zhangi* Tong & Li, 2007 **17***B.zhouxi* sp. nov. **18***B.zunyi* sp. nov.

Guizhou, located in the southwest of China, is renowned for its abundant karst caves. The extreme cave environmental conditions have been regarded as the primary factors of maintaining species endemism within caves (Lefébure et al. 2006; Yao et al. 2016). Nevertheless, only eight endemic species of *Belisana* have been recorded from Guizhou (Chen et al. 2009; Zhang and Peng 2011; Chen et al. 2016; Yang et al. 2023a; Wang et al. 2024). Among these, seven species are collected from caves. This work aims to report the nine new species, a new record found in karst caves (Fig. 1), and to provide an updated list of species of *Belisana* from Guizhou (Table 1).

**Table 1.** A list of species of *Belisana* from Guizhou, China.

No.	Species	Habitat	Reference
1	*B.bijie* sp. nov.	karst cave	this paper
2	*B.daji* Chen, Zhang & Zhu, 2009	karst cave	Chen et al. (2009)
3	*B.douqing* Chen, Zhang & Zhu, 2009	karst cave	Chen et al. (2009)
4	*B.galeiformis* Zhang & Peng, 2011	/	Zhang and Peng (2011)
5	*B.lii* Chen, Yu & Guo, 2016	karst cave	Chen et al. (2016)
6	*B.liupanshui* sp. nov.	karst cave	this paper
7	*B.majiang* sp. nov.	karst cave	this paper
8	*B.nayong* sp. nov.	karst cave	this paper
9	*B.qixingguan* sp. nov.	karst cave	this paper
10	*B.wangchengi* Wang, Yao & Zhang, 2024	karst cave	Wang et al. (2024)
11	*B.xishui* Chen, Zhang & Zhu, 2009	karst cave	Chen et al. (2009)
12	*B.xiuwen* sp. nov.	karst cave	this paper
13	*B.yanhe* Chen, Zhang & Zhu, 2009	karst cave	Chen et al. (2009)
14	*B.yongcong* sp. nov.	karst cave	this paper
15	*B.yuhaoi* Yang & Yao, 2023	karst cave	Yang et al. (2023a)
16	*B.zhangi* Tong & Li, 2007	karst cave	this paper; Tong and Li (2007)
17	*B.zhouxi* sp. nov.	karst cave	this paper
18	*B.zunyi* sp. nov.	karst cave	this paper

## ﻿Materials and methods

Specimens were examined and measured with a Leica M205 C stereomicroscope. Left male palps were photographed. Epigynes were photographed before dissection. Vulvae were photographed after treating them in a 10% warm solution of potassium hydroxide (KOH) to dissolve soft tissues. Images were captured with a Canon EOS 750D wide zoom digital camera (24.2 megapixels) mounted on the stereomicroscope mentioned above and assembled using Helicon Focus v. 3.10.3 image stacking software (Khmelik et al. 2005). Drawings were done with Procreate 5.0.2 (Savage Interactive Pty. Ltd.). All measurements are given in millimeters (mm). Leg measurements are shown as: total length (femur, patella, tibia, metatarsus, tarsus). Leg segments were measured on their dorsal sides. The distribution map was generated with ArcGIS v. 10.2 (ESRI Inc.). The specimens studied are preserved in 75% ethanol and deposited in the Institute of Zoology, Chinese Academy of Sciences (**IZCAS**) in Beijing, China.

Terminology and taxonomic descriptions follow Huber (2005) and Yao et al. (2015). The following abbreviations are used: **aa** = anterior arch, **ALE** = anterior lateral eye, **b** = bulb, **ba** = bulbal apophysis, **da** = distal apophysis, **e** = embolus, **ep** = epigynal pocket, **f** = flap, **L/d** = length/diameter, **pa** = proximo-lateral apophysis, **PME** = posterior median eye, **pp** = pore plate, **pr** = procursus.

## ﻿Taxonomy

**Family Pholcidae C.L. Koch, 1850**

**Subfamily Pholcinae C.L. Koch, 1850**

### ﻿Genus Belisana

Taxon classificationAnimaliaAraneaePholcidae

Thorell, 1898

F2FD569E-A592-550E-BE1D-AB9FDB88657A

#### Type species.

*Belisanatauricornis* Thorell, 1898.

### ﻿ Belisana bijie

Taxon classificationAnimaliaAraneaePholcidae

Wang, S. Li & Yao sp. nov.

0B2A9B44-F3CE-5520-B3C9-80275FF5193E

https://zoobank.org/938091C8-867C-4607-9EB1-9B4BE301F78F

**Figure 2.**
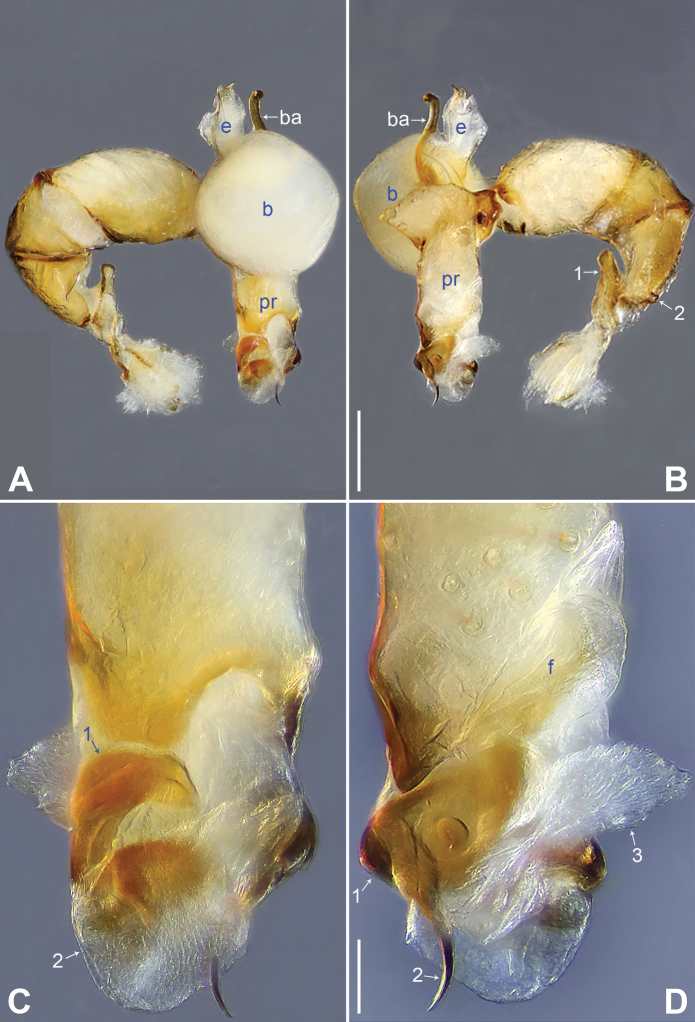
*Belisanabijie* sp. nov., holotype male **A, B** palp (**A** prolateral view **B** retrolateral view, arrow 1 points at ventral apophysis, arrow 2 points at retrolatero-proximal protrusion) **C, D** distal part of procursus (**C** prolateral view, arrow 1 points at prolatero-subdistal sclerite, arrow 2 points at prolatero-distal membranous lamella **D** retrolateral view, arrow 1 points at sclerotized dorso-subdistal apophysis, arrow 2 points at retrolatero-distal spine, arrow 3 points at retrolatero-subdistal membranous process). Abbreviations: b = bulb, ba = bulbal apophysis, e = embolus, f = flap, pr = procursus. Scale bars: 0.20 (**A, B**); 0.05 (**C, D**).

**Figure 3.**
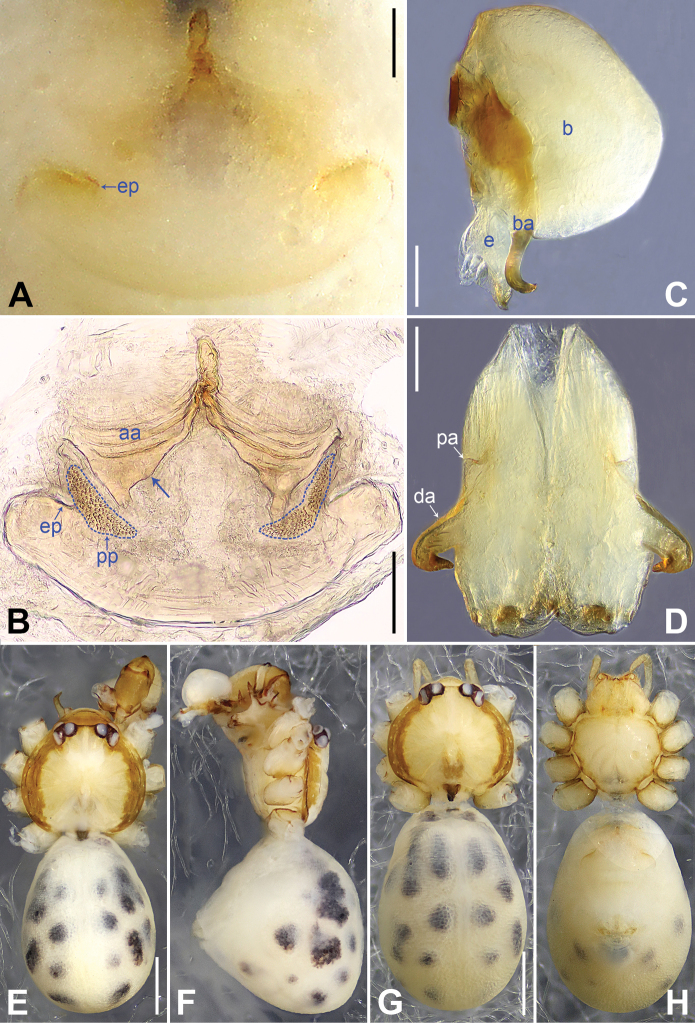
*Belisanabijie* sp. nov., holotype male (**C–F**) and paratype female (**A, B, G, H**) **A** epigyne, ventral view **B** vulva, dorsal view, arrow points at lateral sclerite **C** bulb, prolateral view **D** chelicerae, frontal view **E–H** habitus (**E, G** dorsal view **F** lateral view **H** ventral view). Abbreviations: aa = anterior arch, b = bulb, ba = bulbal apophysis, da = distal apophysis, e = embolus, ep = epigynal pocket, pa = proximo-lateral apophysis, pp = pore plate. Scale bars: 0.10 (**A–D**); 0.40 (**E–H**).

Figs 2 , 3 , 22A, B , 24A, B

#### Type material.

***Holotype*: China** • ♂; Guizhou, Bijie, Qixingguan District, Salaxi Town, Shuanglongdao Cave; 27°11.493'N, 105°03.850'E; alt. 1920 m; 18 Nov. 2011; Z. Chen & Z. Zha leg.; IZCAS-Ar45175. ***Paratype*: China** • 1♀; same data as for holotype; IZCAS-Ar45176.

#### Diagnosis.

The new species resembles *B.wangchengi* Wang, Yao & Zhang, 2024 (Wang et al. 2024: 2, figs 1A–D, 2A–H, 3A–D) by having similar male chelicerae (tips of distal apophyses widely separated and pointing inwards; Fig. 3D), bulbal apophysis (hooked; Fig. 3C), and epigyne (epigynal pockets on lateral part of epigynal plate; Figs 3A, 24A), but can be distinguished by procursus with retrolatero-subdistal membranous process (arrow 3 in Figs 2D, 22B vs absent), by vulval pore plates with nearly angular sclerites (arrow in Figs 3B, 24B vs blunt), and by dorsal shield of prosoma with lateral brown bands (Fig. 3E, G vs absent, but with median radiating marks).

#### Description.

**Male** (**holotype**): Total length 1.95 (2.08 with clypeus), prosoma 0.70 long, 0.82 wide, opisthosoma 1.25 long, 0.90 wide. Leg I: 19.96 (5.13, 0.38, 5.05, 7.52, 1.88), leg II: 13.25 (3.52, 0.37, 3.32, 4.70, 1.34), leg III: 7.81 (2.25, 0.30, 1.90, 2.59, 0.77), leg IV: 9.83 (3.09, 0.30, 2.45, 3.16, 0.83); tibia I L/d: 73. Eye interdistances and diameters: PME–PME 0.11, PME 0.10, PME–ALE 0.02. Sternum width/length: 0.63/0.56. Habitus as in Fig. 3E, F. Dorsal shield of prosoma yellowish, with brown lateral bands; clypeus brown; sternum yellowish. Legs whitish, without darker rings. Opisthosoma yellowish, with black spots. Thoracic furrow absent. Clypeus unmodified. Chelicerae with pair of proximo-lateral apophyses (pa in Fig. 3D) and pair of curved distal apophyses (distance between tips: 0.31; da in Fig. 3D). Palp as in Fig. 2A, B; trochanter with ventral apophysis (2× longer than wide, arrow 1 in Fig. 2B); femur with retrolatero-proximal protrusion (arrow 2 in Fig. 2B); procursus simple proximally but complex distally, with prolatero-subdistal sclerite (arrow 1 in Figs 2C, 22A), prolatero-distal membranous lamella (arrow 2 in Figs 2C, 22A) bearing proximally sclerotized part, sclerotized dorso-subdistal apophysis (arrow 1 in Figs 2D, 22B), retrolatero-distal spine (arrow 2 in Figs 2D, 22B), retrolatero-subdistal membranous process (arrow 3 in Figs 2D, 22B), and retrolateral membranous flap (f in Figs 2D, 22B); bulb with hooked apophysis (ba in Fig. 3C) and simple embolus (e in Fig. 3C). Retrolateral trichobothria on tibia I at 9% proximally; legs with short vertical setae on metatarsi; tarsus I with 17 distinct pseudosegments.

**Female** (**paratype**, IZCAS-Ar45176): Similar to male, habitus as in Fig. 3G, H. Total length 1.96 (2.09 with clypeus), prosoma 0.66 long, 0.83 wide, opisthosoma 1.30 long, 1.01 wide; tibia I: 3.40; tibia I L/d: 49. Eye interdistances and diameters: PME–PME 0.10, PME 0.10, PME–ALE 0.02. Sternum width/length: 0.60/0.53. Dorsal shield of prosoma with distinct postero-median marks; clypeus yellowish. Epigyne simple and flat, posteriorly curved, with pair of lateral pockets 0.31 apart (ep in Figs 3A, 24A). Vulva with ridge-shaped anterior arch bearing pair of nearly angular sclerites (arrow in Figs 3B, 24B), and pair of nearly triangular pore plates (4× longer than wide, pp in Figs 3B, 24B).

#### Habitat.

The species was found in the dark zone inside the cave.

#### Distribution.

China (Guizhou, type locality; Fig. 1).

#### Etymology.

The specific name refers to the type locality; noun in apposition.

### ﻿ Belisana liupanshui

Taxon classificationAnimaliaAraneaePholcidae

Wang, S. Li & Yao sp. nov.

712BAFCC-AD3F-56E5-96F4-90BDE5164A50

https://zoobank.org/4C5D21CD-86E7-43FA-B974-0235DB601B2B

**Figure 4.**
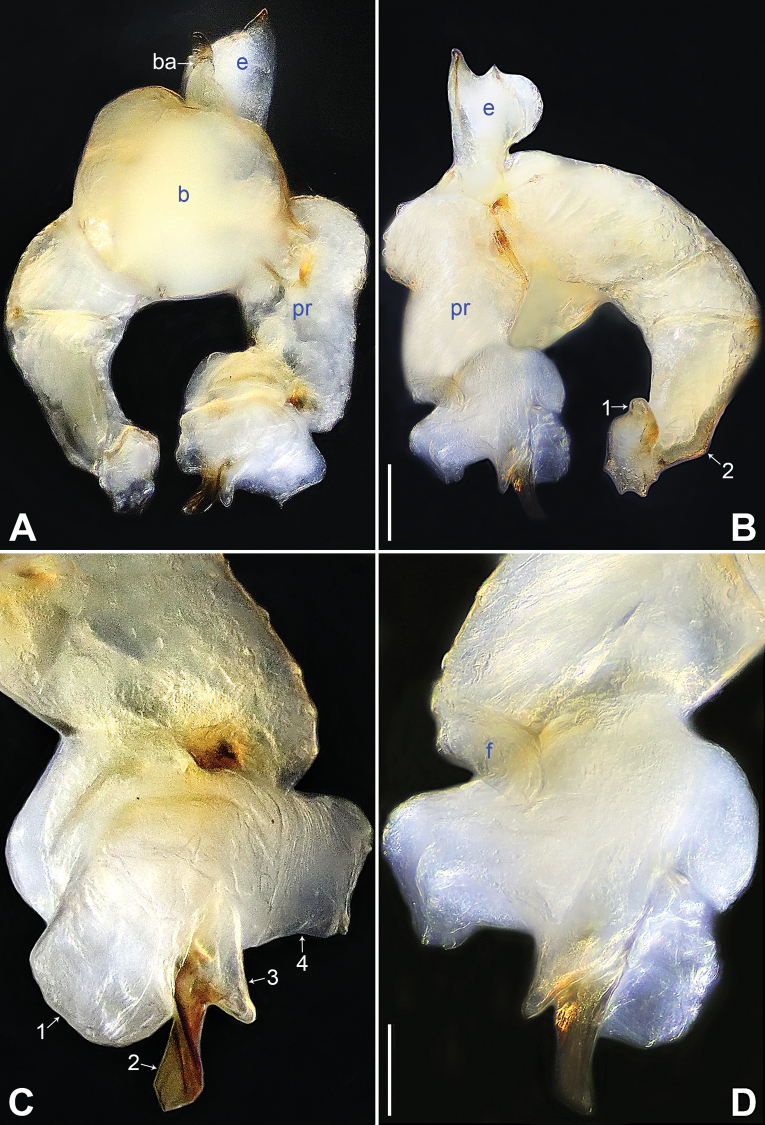
*Belisanaliupanshui* sp. nov., holotype male **A, B** palp (**A** prolateral view **B** retrolateral view, arrow 1 points at ventral apophysis, arrow 2 points at retrolatero-proximal protrusion) **C, D** distal part of procursus (**C** prolateral view, arrow 1 points at prolatero-subdistal membranous process, arrow 2 points at sclerotized distal apophysis, arrow 3 points at subdistal membranous process, arrow 4 points at dorso-subdistal membranous process **D** retrolateral view). Abbreviations: b = bulb, ba = bulbal apophysis, e = embolus, f = flap, pr = procursus. Scale bars: 0.10 (**A, B**); 0.05 (**C, D**).

**Figure 5.**
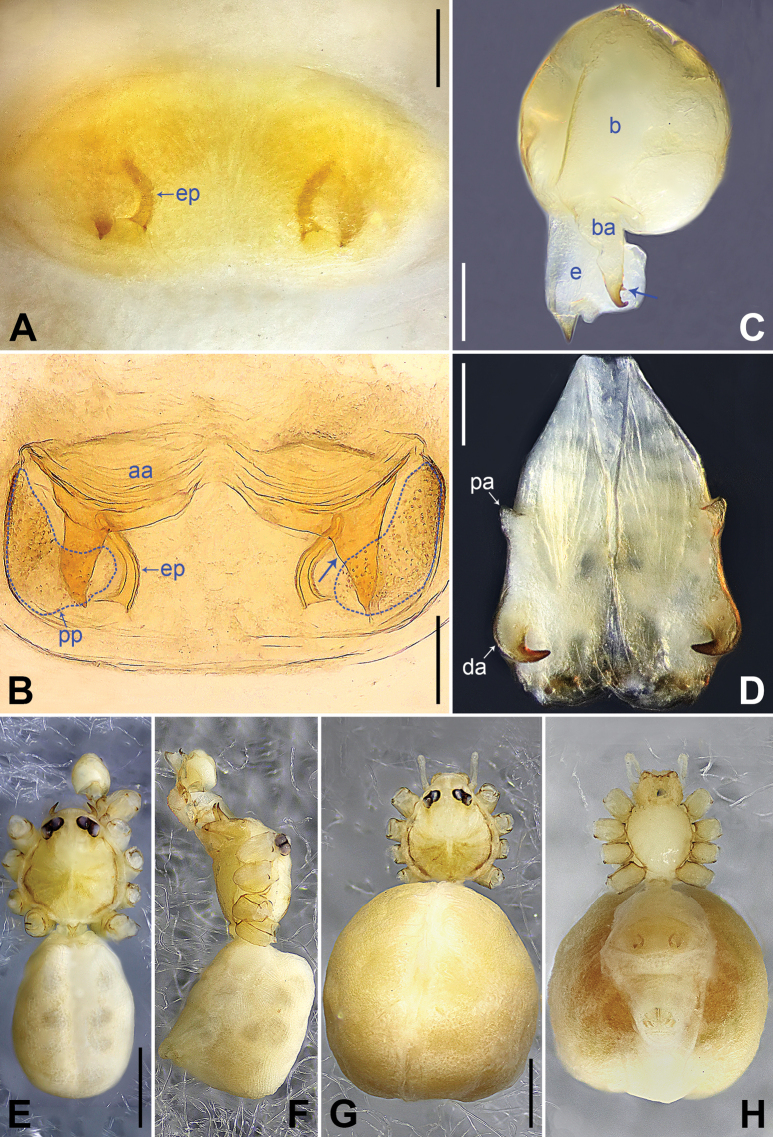
*Belisanaliupanshui* sp. nov., holotype male (**C–F**) and paratype female (**A, B, G, H**) **A** epigyne, ventral view **B** vulva, dorsal view, arrow points at lateral sclerite **C** bulb, prolateral view, arrow points at subdistal apophysis **D** chelicerae, frontal view **E–H** habitus (**E, G** dorsal view **F** lateral view **H** ventral view). Abbreviations: aa = anterior arch, b = bulb, ba = bulbal apophysis, da = distal apophysis, e = embolus, ep = epigynal pocket, pa = proximo-lateral apophysis, pp = pore plate. Scale bars: 0.10 (**A–D**); 0.50 (**E–H**).

Figs 4 , 5 , 22C, D , 24C, D

#### Type material.

***Holotype*: China** • ♂; Guizhou, Liupanshui, Pan County, Biyun Cave; 25°46.527'N, 104°38.278'E; alt. 1468 m; 13 Apr. 2007; J. Liu & Y. Lin leg.; IZCAS-Ar45181. ***Paratypes*: China** • 5♂; same data as for holotype; IZCAS-Ar45182–86 • 7♀; same data as for holotype; IZCAS-Ar45187–93.

#### Diagnosis.

The new species resembles *B.jiuxiang* Zhang, Li & Yao, 2024 (Zhang et al. 2024b: 261, figs 4A–D, 5A–H, 18C, D, 20C, D) by having similar male chelicerae (distal apophyses directed towards frontally, but tips pointing inwards; Fig. 5D) and epigyne (epigynal pockets on median part of epigynal plate, epigynal plate posteriorly straight; Figs 5A, 24C), but can be distinguished by procursus with prolatero-subdistal and subdistal membranous processes (arrows 1, 3 in Figs 4C, 22C vs absent) and nearly rectangle dorso-subdistal membranous process (arrow 4 in Figs 4C, 22C vs angular), by bulbal apophysis with angular subdistal apophysis (arrow in Fig. 5C vs absent), and by vulval pore plates anteriorly narrow and posteriorly wide (pp in Figs 5B, 24D vs nearly quadrilateral).

#### Description.

**Male** (**holotype**): Total length 1.71 (1.79 with clypeus), prosoma 0.63 long, 0.67 wide, opisthosoma 1.08 long, 0.81 wide. Leg I: – (4.04, 0.29, 4.50, 5.96, –), leg II: 11.08 (2.80, 0.30, 2.97, 3.92, 1.09), leg III: 7.98 (2.22, 0.25, 1.98, 2.72, 0.81), leg IV: 10.20 (3.00, 0.26, 2.63, 3.45, 0.86); tibia I L/d: 63. Eye interdistances and diameters: PME–PME 0.09, PME 0.08, PME–ALE 0.02. Sternum width/length: 0.51/0.44. Habitus as in Fig. 5E, F. Dorsal shield of prosoma yellowish, with brownish radiating marks; clypeus brownish; sternum yellowish. Legs whitish, without darker rings. Opisthosoma yellowish, without spots. Thoracic furrow absent. Clypeus unmodified. Chelicerae with pair of proximo-lateral apophyses (pa in Fig. 5D) and pair of curved distal apophyses (distance between tips: 0.17; da in Fig. 5D). Palp as in Fig. 4A, B; trochanter with ventral apophysis (as long as wide, arrow 1 in Fig. 4B); femur with retrolatero-proximal protrusion (arrow 2 in Fig. 4B); procursus simple proximally but complex distally, with prolatero-subdistal membranous process (arrow 1 in Figs 4C, 22C), sclerotized distal apophysis (arrow 2 in Figs 4C, 22C), subdistal membranous process (arrow 3 in Figs 4C, 22C), dorso-subdistal membranous process (arrow 4 in Figs 4C, 22C), and retrolateral membranous flap (f in Figs 4D, 22D); bulb with hooked apophysis bearing angular subdistal apophysis (arrow in Fig. 5C) and simple embolus (e in Fig. 5C). Retrolateral trichobothria on tibia I at 4% proximally; legs with short vertical setae on metatarsi.

**Female** (**paratype**, IZCAS-Ar45187): Similar to male, habitus as in Fig. 5G, H. Total length 2.53 (2.65 with clypeus), prosoma 0.67 long, 0.75 wide, opisthosoma 1.86 long, 1.80 wide; tibia I: 4.05; tibia I L/d: 51. Eye interdistances and diameters: PME–PME 0.11, PME 0.07, PME–ALE 0.02. Sternum width/length: 0.52/0.46. Epigyne simple and flat, posteriorly straight, with pair of median pockets 0.20 apart (ep in Figs 5A, 24C). Vulva with ridge-shaped anterior arch bearing pair of angular lateral sclerites (arrow in Figs 5B, 24D), and pair of anteriorly narrow and posteriorly wide pore plates (3× longer than wide, pp in Figs 5B, 24D).

#### Variation.

Tibia I in five male paratypes (IZCAS-Ar45182–86): 4.50, 4.55, 4.62, 4.65, 4.70. Tibia I in the other six female paratypes (IZCAS-Ar45188–93): 3.56–3.85.

#### Habitat.

The species was found in the dark zone inside the cave.

#### Distribution.

China (Guizhou, type locality; Fig. 1).

#### Etymology.

The specific name refers to the type locality; noun in apposition.

### ﻿ Belisana majiang

Taxon classificationAnimaliaAraneaePholcidae

Wang, S. Li & Yao sp. nov.

6422EB57-54B0-5143-A219-5D60B09B121B

https://zoobank.org/75C0DB39-2F6A-40D1-93A0-3C85CD956A53

**Figure 6.**
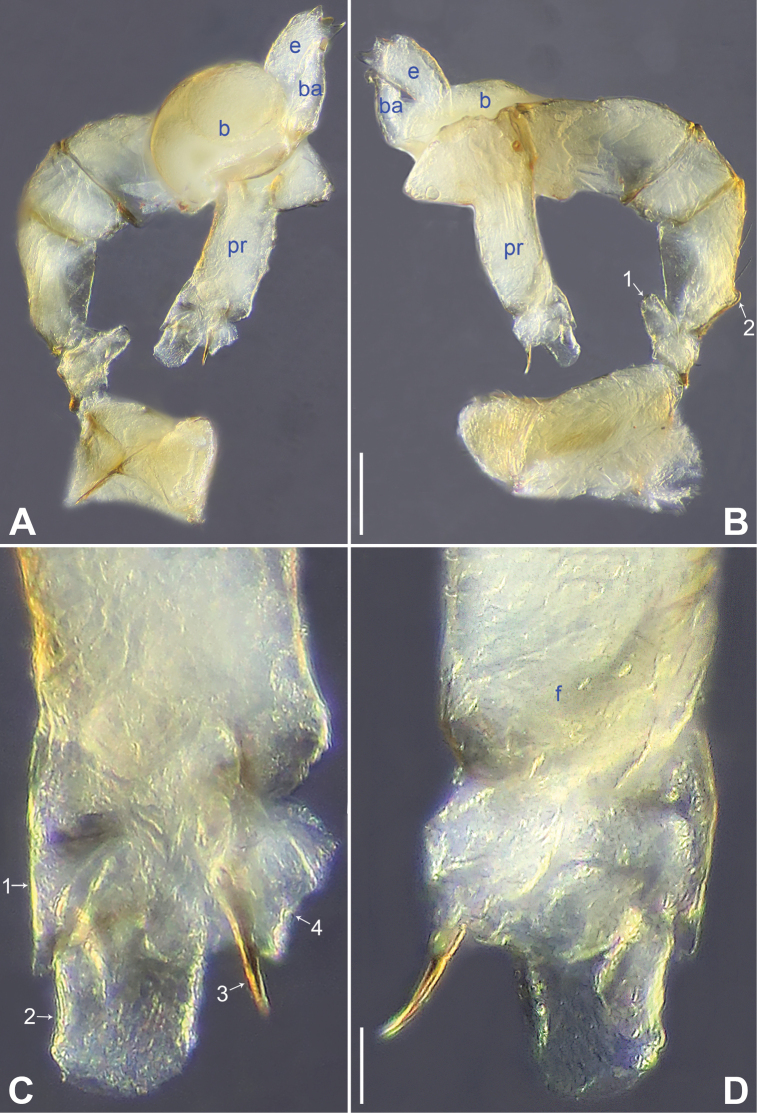
*Belisanamajiang* sp. nov., holotype male **A, B** palp (**A** prolateral view **B** retrolateral view, arrow 1 points at ventral apophysis, arrow 2 points at retrolatero-proximal protrusion) **C, D** distal part of procursus (**C** prolateral view, arrow 1 points at ventro-subdistal membranous process, arrow 2 points at prolatero-distal membranous lamella, arrow 3 points at dorso-distal spine, arrow 4 points at dorso-subdistal membranous process **D** retrolateral view). Abbreviations: b = bulb, ba = bulbal apophysis, e = embolus, f = flap, pr = procursus. Scale bars: 0.10 (**A, B**); 0.02 (**C, D**).

**Figure 7.**
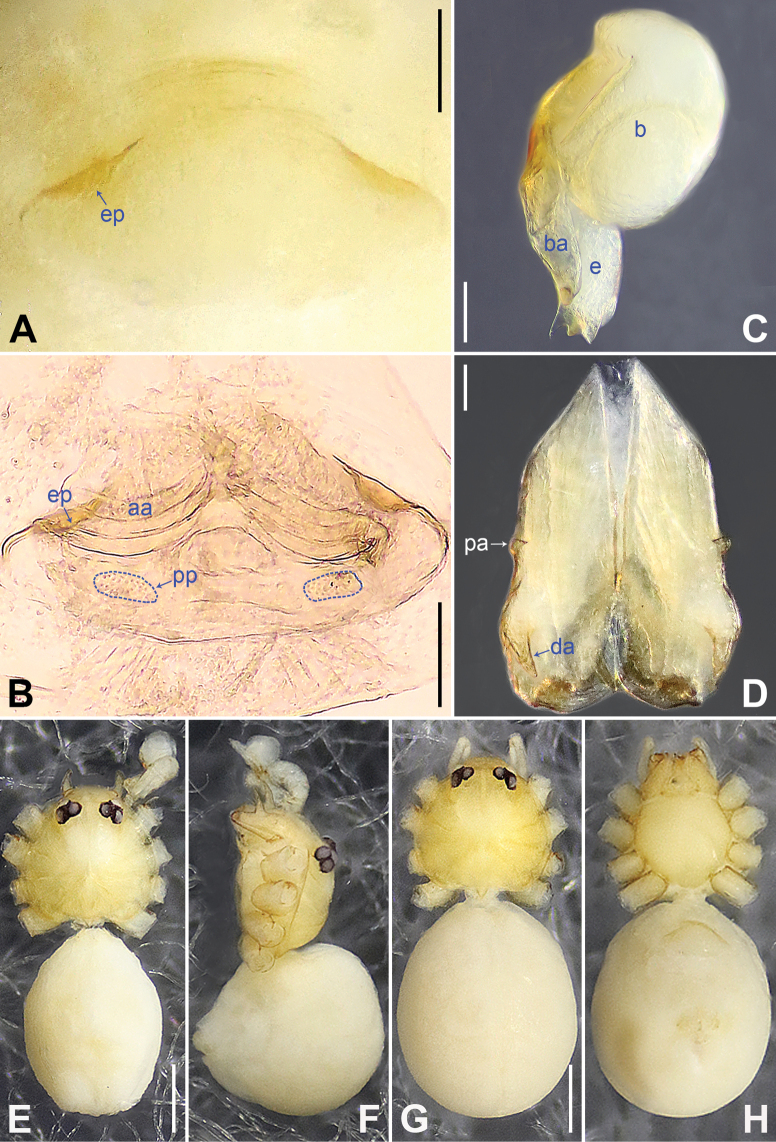
*Belisanamajiang* sp. nov., holotype male (**C–F**) and paratype female (**A, B, G, H**) **A** epigyne, ventral view **B** vulva, dorsal view **C** bulb, prolateral view **D** chelicerae, frontal view **E–H** habitus (**E, G** dorsal view **F** lateral view **H** ventral view). Abbreviations: aa = anterior arch, b = bulb, ba = bulbal apophysis, da = distal apophysis, e = embolus, ep = epigynal pocket, pa = proximo-lateral apophysis, pp = pore plate. Scale bars: 0.05 (**A–D**); 0.30 (**E–H**).

Figs 6 , 7 , 22E, F , 24E, F

#### Type material.

***Holotype*: China** • ♂; Guizhou, Kaili, Majiang County, Xingshan Town, Gubin Village, Guazhutou Cave; 26°30.257'N, 107°30.943'E; alt. 1056 m; 28 Nov. 2011; Z. Chen & Z. Zha leg.; IZCAS-Ar45194. ***Paratypes*: China** • 1♂; same data as for holotype; IZCAS-Ar45195 • 2♀; same data as for holotype; IZCAS-Ar45196–97.

#### Diagnosis.

The new species resembles *B.zhouxi* sp. nov. (Figs 18, 19, 23E, F, 25G, H) by having similar male chelicerae (tips of distal apophyses pointing downwards; Fig. 7D), bulbal apophysis (hooked; Fig. 7C), and epigyne (epigynal pockets on antero-lateral part of epigynal plate, epigynal plate posteriorly curved; Figs 7A, 24E), but can be distinguished by procursus with distinct ventro-subdistal membranous process and dorso-distal spine (arrows 1, 3 in Figs 6C, 22E vs absent) and by vulval pore plates long elliptic (3× longer than wide, pp in Figs 7B, 24F vs 2×); also distinguished from *B.tongle* Zhang, Chen & Zhu, 2008 (Zhang et al. 2008: 654, figs 1–5) by procursus with dorso-subdistal membranous process (arrow 4 in Figs 6C, 22E vs absent), by prolatero-distal membranous lamella of procursus without rectangular sclerite (arrow 2 in Figs 6C, 22E vs present), by ventro-subdistal membranous process of procursus 3× longer than wide (arrow 1 in Figs 6C, 22E vs 8×), and by dorso-distal spine of procursus 6× longer than wide (arrow 3 in Figs 6C, 22E vs 12×).

#### Description.

**Male** (**holotype**): Total length 1.35 (1.43 with clypeus), prosoma 0.52 long, 0.59 wide, opisthosoma 0.83 long, 0.64 wide. Leg I missing, leg II: 6.13 (1.78, 0.22, 1.50, 1.90, 0.73), leg III: 4.19 (1.28, 0.20, 0.95, 1.33, 0.43), leg IV: 5.87 (1.72, 0.22, 1.48, 1.80, 0.65). Eye interdistances and diameters: PME–PME 0.08, PME 0.06, PME–ALE 0.02. Sternum width/length: 0.44/0.38. Habitus as in Fig. 7E, F. Dorsal shield of prosoma yellowish, without marks; clypeus and sternum yellowish. Legs whitish, without darker rings. Opisthosoma yellowish, without spots. Thoracic furrow absent. Clypeus unmodified. Chelicerae with pair of proximo-lateral apophyses (pa in Fig. 7D) and pair of curved distal apophyses (distance between tips: 0.19; da in Fig. 7D). Palp as in Fig. 6A, B; trochanter with ventral apophysis (1.5× longer than wide, arrow 1 in Fig. 6B); femur with retrolatero-proximal protrusion (arrow 2 in Fig. 6B); procursus simple proximally but complex distally, with ventro-subdistal membranous process (arrow 1 in Figs 6C, 22E), prolatero-distal membranous lamella (arrow 2 in Figs 6C, 22E) bearing proximally slightly sclerotized part, dorso-distal spine (arrow 3 in Figs 6C, 22E), dorso-subdistal membranous process (arrow 4 in Figs 6C, 22E), and retrolateral membranous flap (f in Figs 6D, 22F); bulb with hooked apophysis (ba in Fig. 7C) and simple embolus (e in Fig. 7C).

**Female** (**paratype**, IZCAS-Ar45196): Similar to male, habitus as in Fig. 7G, H. Total length 1.48 (1.58 with clypeus), prosoma 0.48 long, 0.56 wide, opisthosoma 1.00 long, 0.84 wide. Eye interdistances and diameters: PME–PME 0.08, PME 0.06, PME–ALE 0.02. Sternum width/length: 0.43/0.39. Epigyne simple and flat, posteriorly curved, with pair of antero-lateral pockets 0.14 apart (ep in Figs 7A, 24E). Vulva with ridge-shaped anterior arch (aa in Figs 7B, 24F) and pair of long elliptic pore plates (3× longer than wide, pp in Figs 7B, 24F).

#### Variation.

Unknown. Legs I missing in male paratype (IZCAS-Ar45195) and another female paratype (IZCAS-Ar45197).

#### Habitat.

The species was found in the dark zone inside the cave.

#### Distribution.

China (Guizhou, type locality; Fig. 1).

#### Etymology.

The specific name refers to the type locality; noun in apposition.

### ﻿ Belisana nayong

Taxon classificationAnimaliaAraneaePholcidae

Wang, S. Li & Yao sp. nov.

C614FD4B-0E6A-5C84-A58B-BE06859E2EEA

https://zoobank.org/2374804C-1CDB-4013-885E-336618ED0D2B

**Figure 8.**
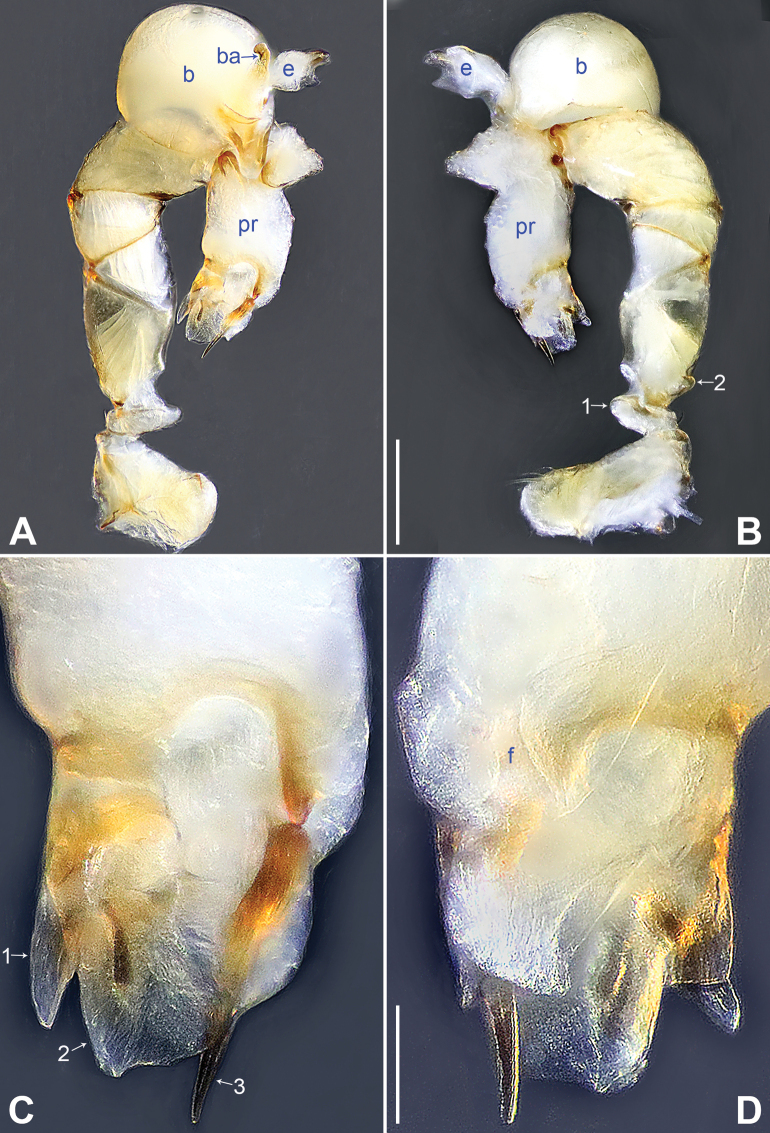
*Belisananayong* sp. nov., holotype male **A, B** palp (**A** prolateral view **B** retrolateral view, arrow 1 points at ventral apophysis, arrow 2 points at retrolatero-proximal protrusion) **C, D** distal part of procursus (**C** prolateral view, arrow 1 points at ventro-subdistal membranous process, arrow 2 points at prolatero-distal membranous lamella, arrow 3 points at dorso-distal spine **D** retrolateral view). Abbreviations: b = bulb, ba = bulbal apophysis, e = embolus, f = flap, pr = procursus. Scale bars: 0.20 (**A, B**); 0.05 (**C, D**).

**Figure 9.**
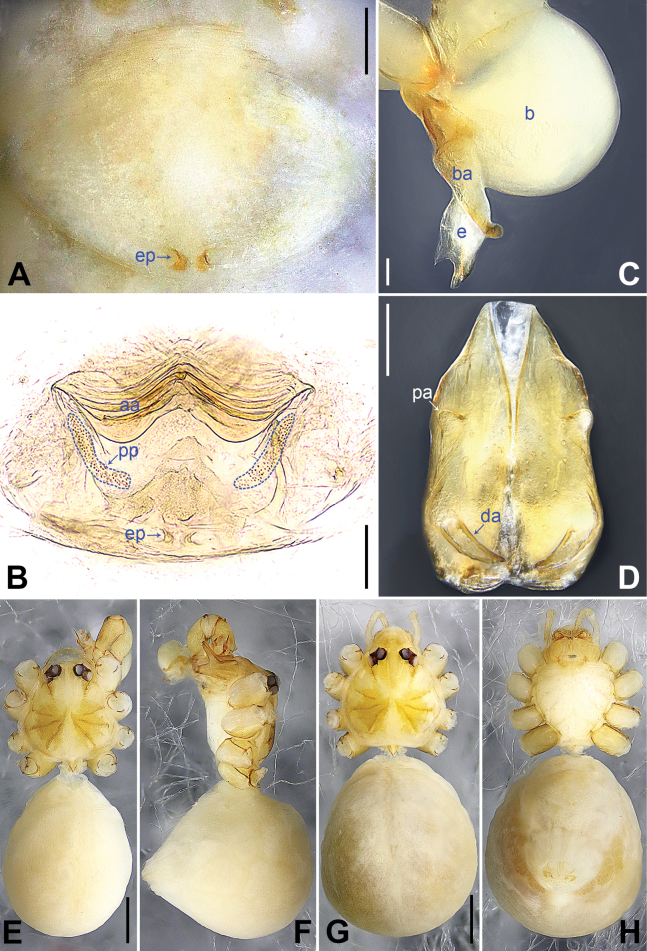
*Belisananayong* sp. nov., holotype male (**C–F**) and paratype female (**A, B, G, H**) **A** epigyne, ventral view **B** vulva, dorsal view **C** bulb, prolateral view **D** chelicerae, frontal view **E–H** habitus (**E, G** dorsal view **F** lateral view **H** ventral view). Abbreviations: aa = anterior arch, b = bulb, ba = bulbal apophysis, da = distal apophysis, e = embolus, ep = epigynal pocket, pa = proximo-lateral apophysis, pp = pore plate. Scale bars: 0.05 (**A–D**); 0.40 (**E–H**).

Figs 8 , 9 , 22G, H , 24G, H

#### Type material.

***Holotype*: China** • ♂; Guizhou, Bijie, Nayong County, Laosiba Town, Bailong Cave; 26°50.166'N, 105°31.222'E; alt. 1468 m; 27 Apr. 2007; J. Liu & Y. Lin leg.; IZCAS-Ar45198. ***Paratypes*: China** • 3♂; same data as for holotype; IZCAS-Ar45199–201 • 4♀; same data as for holotype; IZCAS-Ar45202–05.

#### Diagnosis.

The new species resembles *B.yongcong* sp. nov. (Figs 14, 15, 23A, B, 25C, D) by having similar bulbal apophysis (hooked; Fig. 9C) and vulva (anterior arch ridge-shaped, pore plates curved, long elliptic and 8× longer than wide; Figs 9B, 24H), but can be distinguished by procursus with pointed ventro-subdistal membranous process (arrow 1 in Figs 8C, 22G vs blunt), by male cheliceral distal apophyses pointing inwards (da in Fig. 9D vs outwards), and by epigyne with postero-median pockets (ep in Figs 9A, 24G vs lateral).

#### Description.

**Male** (**holotype**): Total length 2.33 (2.50 with clypeus), prosoma 0.86 long, 0.89 wide, opisthosoma 1.47 long, 1.15 wide. Leg I: 31.51 (8.33, 0.42, 7.82, 13.08, 1.86), leg II missing, leg III: 19.77 (5.08, 0.39, 5.04, 7.88, 1.38), leg IV missing; tibia I L/d: 89. Eye interdistances and diameters: PME–PME 0.10, PME 0.10, PME–ALE 0.02. Sternum width/length: 0.68/0.64. Habitus as in Fig. 9E, F. Dorsal shield of prosoma yellowish, with brown radiating marks; clypeus brown; sternum yellowish. Legs whitish, without darker rings. Opisthosoma yellowish, without spots. Thoracic furrow absent. Clypeus unmodified. Chelicerae with pair of proximo-lateral apophyses (pa in Fig. 9D) and pair of curved distal apophyses (distance between tips: 0.02; da in Fig. 9D). Palp as in Fig. 8A, B; trochanter with ventral apophysis (as long as wide, arrow 1 in Fig. 8B); femur with retrolatero-proximal protrusion (arrow 2 in Fig. 8B); procursus simple proximally but complex distally, with ventro-subdistal membranous process (arrow 1 in Figs 8C, 22G), prolatero-distal membranous lamella (arrow 2 in Figs 8C, 22G) bearing proximally sclerotized part, dorso-distal spine (arrow 3 in Figs 8C, 22G), and retrolateral membranous flap (f in Figs 8D, 22H); bulb with hooked apophysis (ba in Fig. 9C) and simple embolus (e in Fig. 9C). Retrolateral trichobothria on tibia I at 4% proximally; legs with short vertical setae on metatarsi; tarsus I with 18 distinct pseudosegments.

**Female** (**paratype**, IZCAS-Ar45202): Similar to male, habitus as in Fig. 9G, H. Total length 2.47 (2.67 with clypeus), prosoma 0.81 long, 0.88 wide, opisthosoma 1.66 long, 1.40 wide; tibia I: 4.65; tibia I L/d: 58. Eye interdistances and diameters: PME–PME 0.11, PME 0.10, PME–ALE 0.02. Sternum width/length: 0.68/0.64. Epigyne simple and flat, posteriorly curved, with pair of postero-median pockets 0.02 apart (ep in Figs 9A, 24G). Vulva with ridge-shaped anterior arch (aa in Figs 9B, 24H) and pair of curved, long elliptic pore plates (8× longer than wide, pp in Figs 9B, 24H).

#### Variation.

Tibia I in three male paratypes (IZCAS-Ar45199–201): 6.15, 6.35, 6.50. Tibia I in the other three female paratypes (IZCAS-Ar45203–05): 4.45, 4.50, 4.74.

#### Habitat.

The species was found in the dark zone inside the cave.

#### Distribution.

China (Guizhou, type locality; Fig. 1).

#### Etymology.

The specific name refers to the type locality; noun in apposition.

### ﻿ Belisana qixingguan

Taxon classificationAnimaliaAraneaePholcidae

Wang, S. Li & Yao sp. nov.

12EF7C27-F270-5B43-803B-632567E519EA

https://zoobank.org/36AE89A6-13DA-416B-9C06-C86DD3300EC4

**Figure 10.**
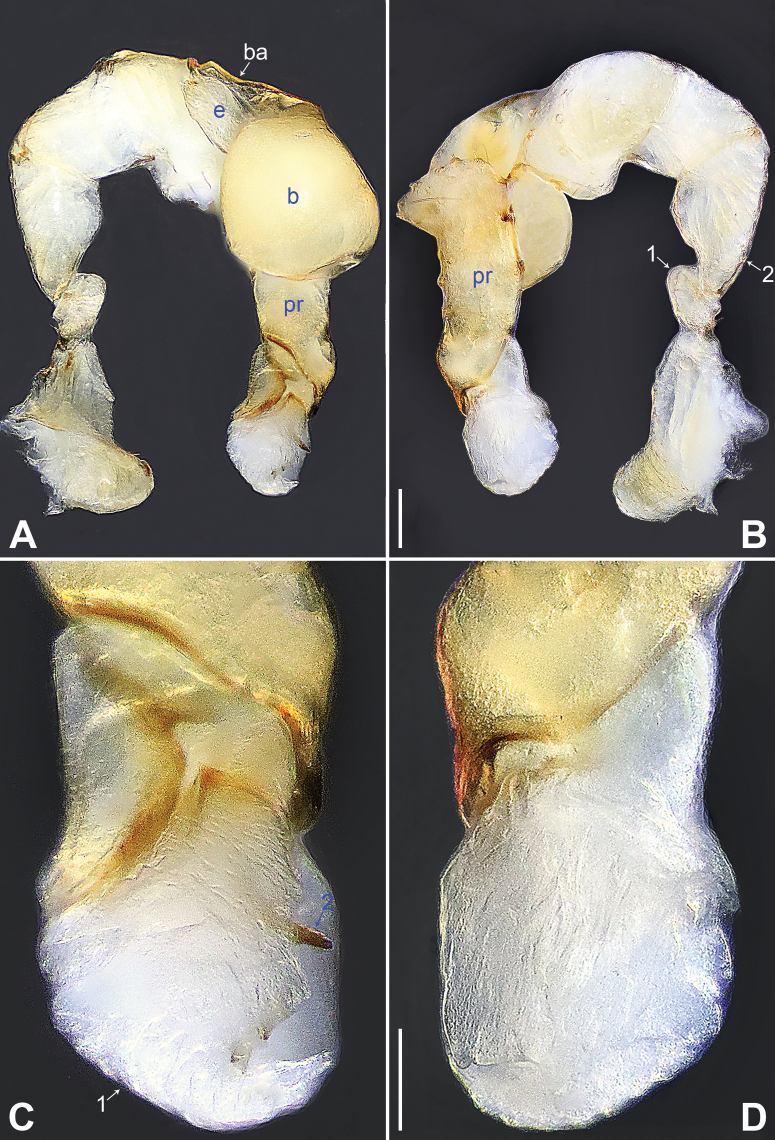
*Belisanaqixingguan* sp. nov., holotype male **A, B** palp (**A** prolateral view **B** retrolateral view, arrow 1 points at ventral apophysis, arrow 2 points at retrolatero-proximal protrusion) **C, D** distal part of procursus (**C** prolateral view, arrow 1 points at distal membranous process, arrow 2 points at subdistal spine **D** retrolateral view). Abbreviations: b = bulb, ba = bulbal apophysis, e = embolus, pr = procursus. Scale bars: 0.10 (**A, B**); 0.05 (**C, D**).

**Figure 11.**
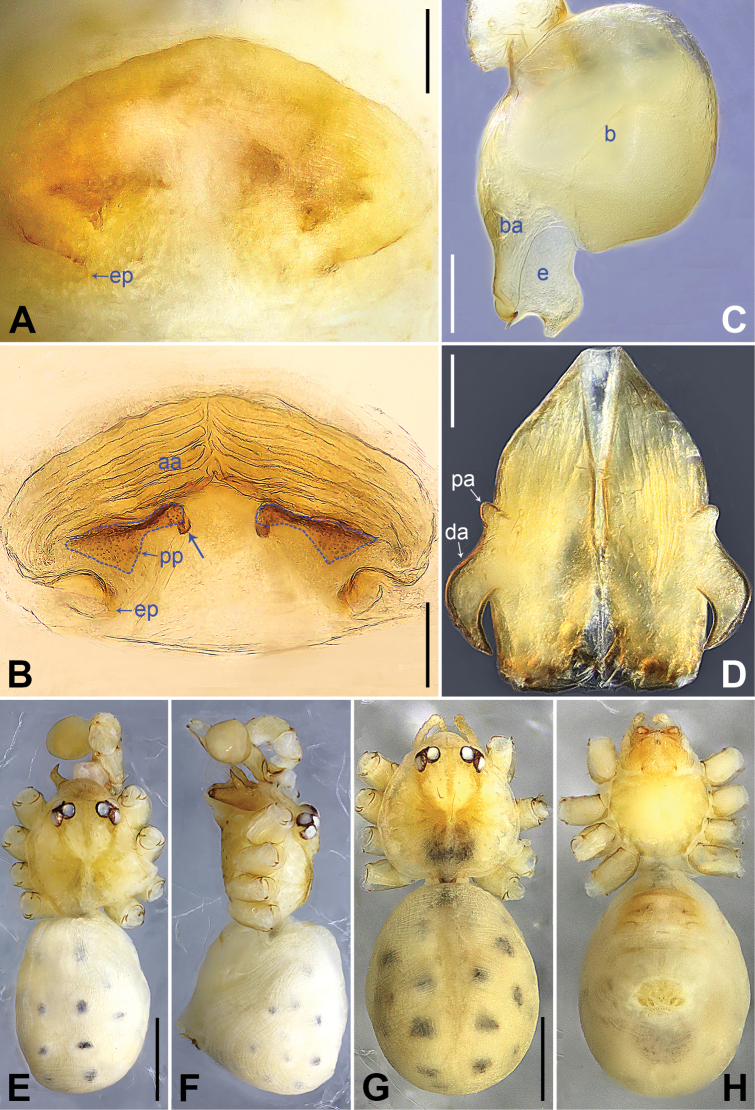
*Belisanaqixingguan* sp. nov., holotype male (**C–F**) and paratype female (**A, B, G, H**) **A** epigyne, ventral view **B** vulva, dorsal view, arrow points at sclerotized protrusion **C** bulb, prolateral view **D** chelicerae, frontal view **E–H** habitus (**E, G** dorsal view **F** lateral view **H** ventral view). Abbreviations: aa = anterior arch, b = bulb, ba = bulbal apophysis, da = distal apophysis, e = embolus, ep = epigynal pocket, pa = proximo-lateral apophysis, pp = pore plate. Scale bars: 0.10 (**A–D**); 0.50 (**E–H**).

Figs 10 , 11 , 22I, J , 24I, J

#### Type material.

***Holotype*: China** • ♂; Guizhou, Bijie, Qixingguan District, Changchun Town, Changchun Village, Changchun Cave; 27°13.904'N, 105°10.397'E; alt. 1580 m; 29 Apr. 2007; J. Liu & Y. Lin leg.; IZCAS-Ar45206. ***Paratypes*: China** • 7♀; same data as for holotype; IZCAS-Ar45207–13.

#### Diagnosis.

The new species resembles *B.tongi* Zhang, Li & Yao, 2024 (Zhang et al. 2024b: 273, figs 12A–D, 13A–H, 18K, L, 21C, D) by having similar distal part of procursus (distal membranous process nearly half-round; arrow 1 in Figs 10C, 22I), bulbal apophysis (hooked; Fig. 11C), and epigyne (epigynal pockets on postero-lateral part of epigynal plate, epigynal plate posteriorly curved; Figs 11A, 24I), but can be distinguished by procursus without distal membranous lamella (Figs 10C, 22I vs present), by male cheliceral distal apophyses pointing downwards (da in Fig. 11D vs outwards), by vulva without teeth (Figs 11B, 24J vs present), and by vulval pore plates medially narrow and posteriorly strongly widened (pp in Figs 11B, 24J vs nearly triangular).

**Figure 12.**
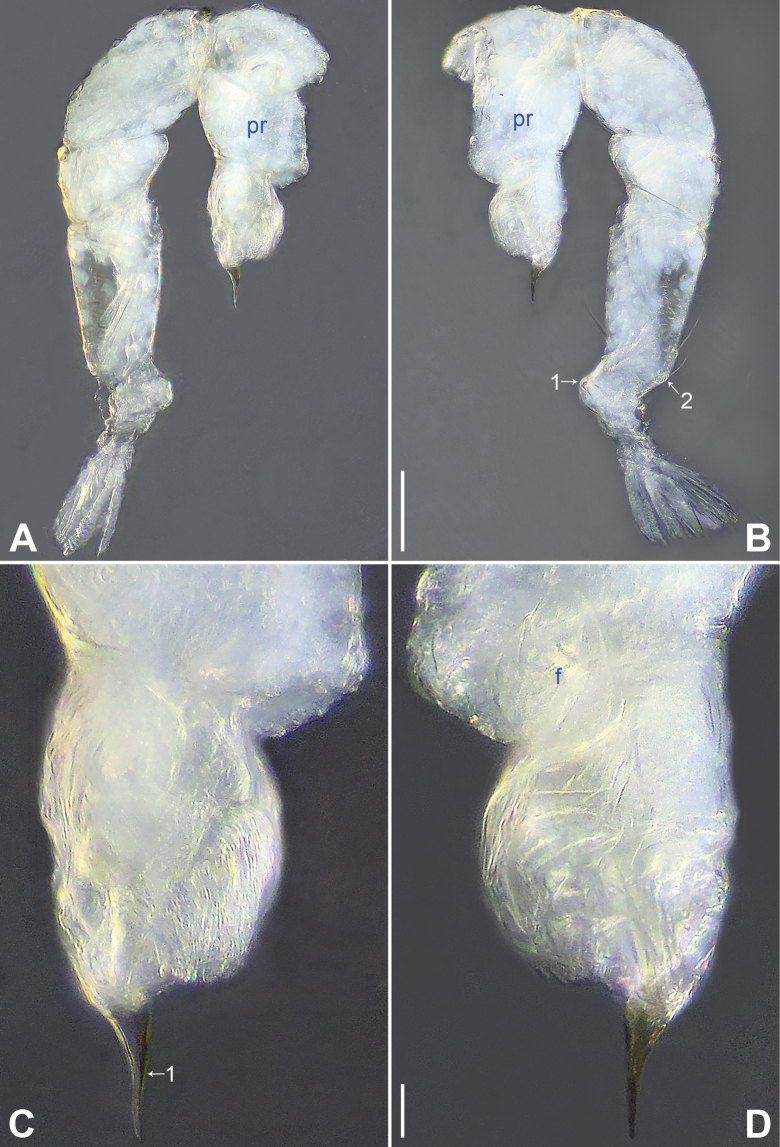
*Belisanaxiuwen* sp. nov., holotype male **A, B** palp (**A** prolateral view **B** retrolateral view, arrow 1 points at ventral apophysis, arrow 2 points at retrolatero-proximal protrusion) **C, D** distal part of procursus (**C** prolateral view, arrow 1 points at distal spine **D** retrolateral view). Abbreviations: f = flap, pr = procursus. Scale bars: 0.10 (**A, B**); 0.02 (**C, D**).

**Figure 13.**
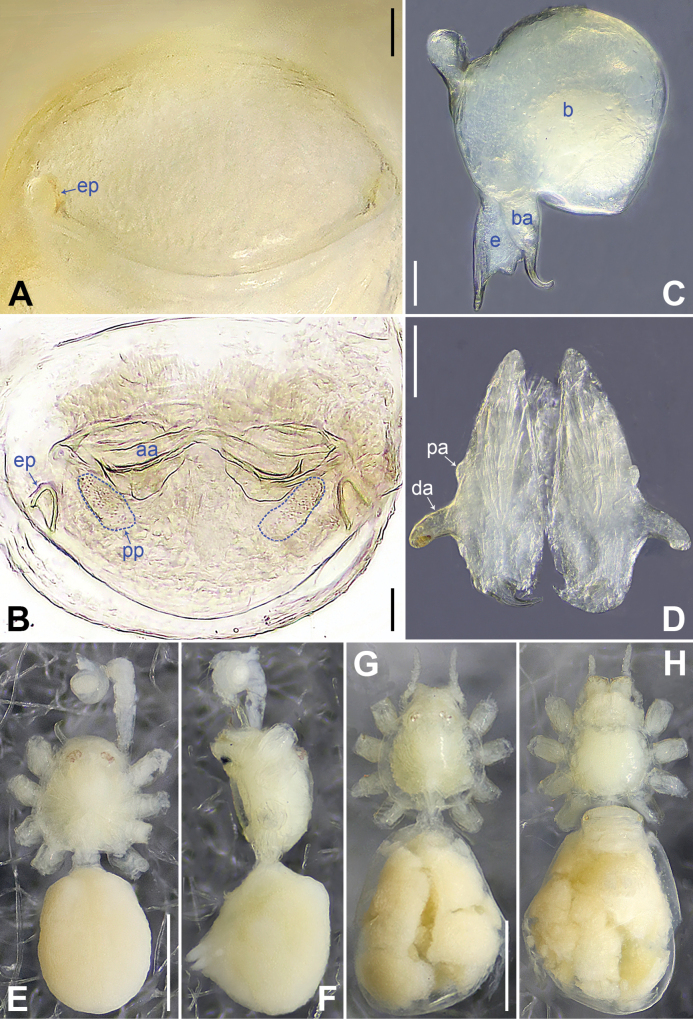
*Belisanaxiuwen* sp. nov., holotype male (**C–F**) and paratype female (**A, B, G, H**) **A** epigyne, ventral view **B** vulva, dorsal view **C** bulb, prolateral view **D** chelicerae, frontal view **E–H** habitus (**E, G** dorsal view **F** lateral view **H** ventral view). Abbreviations: aa = anterior arch, b = bulb, ba = bulbal apophysis, da = distal apophysis, e = embolus, ep = epigynal pocket, pa = proximo-lateral apophysis, pp = pore plate. Scale bars: 0.05 (**A–D**); 0.50 (**E–H**).

#### Description.

**Male** (**holotype**): Total length 1.78 (1.89 with clypeus), prosoma 0.68 long, 0.75 wide, opisthosoma 1.10 long, 0.77 wide. Leg I: 14.43 (3.80, 0.31, 3.72, 5.00, 1.60), leg II: – (3.00, 0.31, 2.66, 3.75, –), leg III missing, leg IV: 7.17 (2.13, 0.27, 1.66, 2.41, 0.70); tibia I L/d: 50. Eye interdistances and diameters: PME–PME 0.14, PME 0.08, PME–ALE 0.02. Sternum width/length: 0.55/0.48. Habitus as in Fig. 11E, F. Dorsal shield of prosoma yellowish, with brown radiating marks; ocular area with brown median stripe; clypeus brown; sternum yellowish. Legs whitish, without darker rings. Opisthosoma yellowish, with black spots. Thoracic furrow absent. Clypeus unmodified. Chelicerae with pair of proximo-lateral apophyses (pa in Fig. 11D) and pair of curved distal apophyses (distance between tips: 0.29; da in Fig. 11D). Palp as in Fig. 10A, B; trochanter with ventral apophysis (as long as wide, arrow 1 in Fig. 10B); femur with retrolatero-proximal protrusion (arrow 2 in Fig. 10B); procursus simple, with distal membranous process (arrow 1 in Figs 10C, 22I) and subdistal spine (arrow 2 in Figs 10C, 22I); bulb with hooked apophysis (ba in Fig. 11C) and simple embolus (e in Fig. 11C). Retrolateral trichobothria on tibia I at 10% proximally; legs with short vertical setae on metatarsi; tarsus I with 15 distinct pseudosegments.

**Female** (**paratype**, IZCAS-Ar45207): Similar to male, habitus as in Fig. 11G, H. Total length 2.04 (2.15 with clypeus), prosoma 0.78 long, 0.81 wide, opisthosoma 1.26 long, 1.00 wide; tibia I: 3.08; tibia I L/d: 39. Eye interdistances and diameters: PME–PME 0.12, PME 0.08, PME–ALE 0.02. Sternum width/length: 0.55/0.46. Epigyne simple and flat, posteriorly curved, with pair of postero-lateral pockets 0.28 apart (ep in Figs 11A, 24I). Vulva with curved anterior arch (aa in Figs 11B, 24J), pair of medially narrow and posteriorly strongly widened pore plates (pp in Figs 11B, 24J), and pair of sclerotized protrusions (arrow in Figs 11B, 24J).

#### Variation.

Tibia I in the other six female paratypes (IZCAS-Ar45208–13): 2.89–3.09.

#### Habitat.

The species was found in the dark zone inside the cave.

#### Distribution.

China (Guizhou, type locality; Fig. 1).

#### Etymology.

The specific name refers to the type locality; noun in apposition.

### ﻿ Belisana xiuwen

Taxon classificationAnimaliaAraneaePholcidae

Wang, S. Li & Yao sp. nov.

39A5A391-0720-5634-AF79-DE26FEAC4E47

https://zoobank.org/92A64C94-8BCB-4211-9EDE-4930EEC2467A

Figs 12 , 13 , 22K, L , 25A, B

#### Type material.

***Holotype*: China** • ♂; Guizhou, Guiyang, Xiuwen County, Liutong Town, Daxing Village, Duobing Cave; 27°06.552'N, 106°29.675'E; alt. 1035 m; 20 Apr. 2007; J. Liu & Y. Lin leg.; IZCAS-Ar45214. ***Paratypes*: China** • 3♀; same data as for holotype; IZCAS-Ar45215–17.

#### Diagnosis.

**Figure 14.**
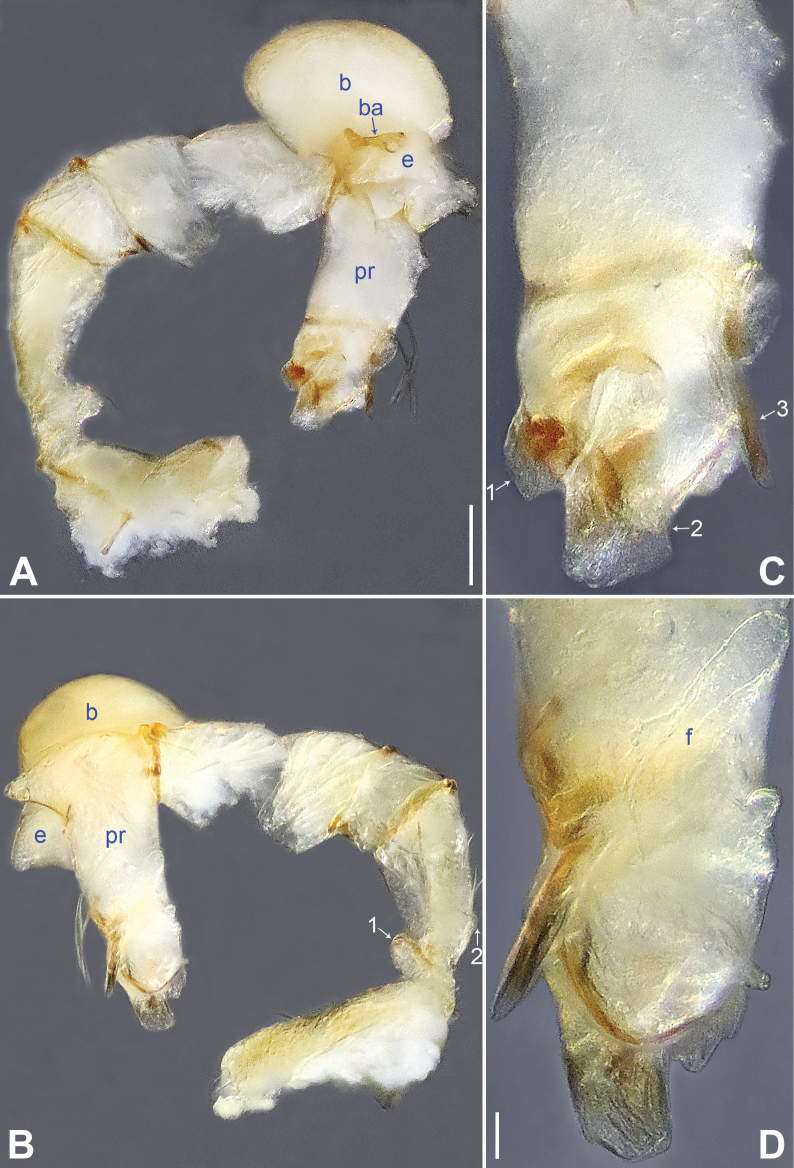
*Belisanayongcong* sp. nov., holotype male **A, B** palp (**A** prolateral view **B** retrolateral view, arrow 1 points at ventral apophysis, arrow 2 points at retrolatero-proximal protrusion) **C, D** distal part of procursus (**C** prolateral view, arrow 1 points at ventro-subdistal membranous lamella, arrow 2 points at prolatero-distal membranous lamella, arrow 3 points at sclerotized dorso-subdistal apophysis **D** retrolateral view). Abbreviations: b = bulb, ba = bulbal apophysis, e = embolus, f = flap, pr = procursus. Scale bars: 0.10 (**A, B**); 0.02 (**C, D**).

**Figure 15.**
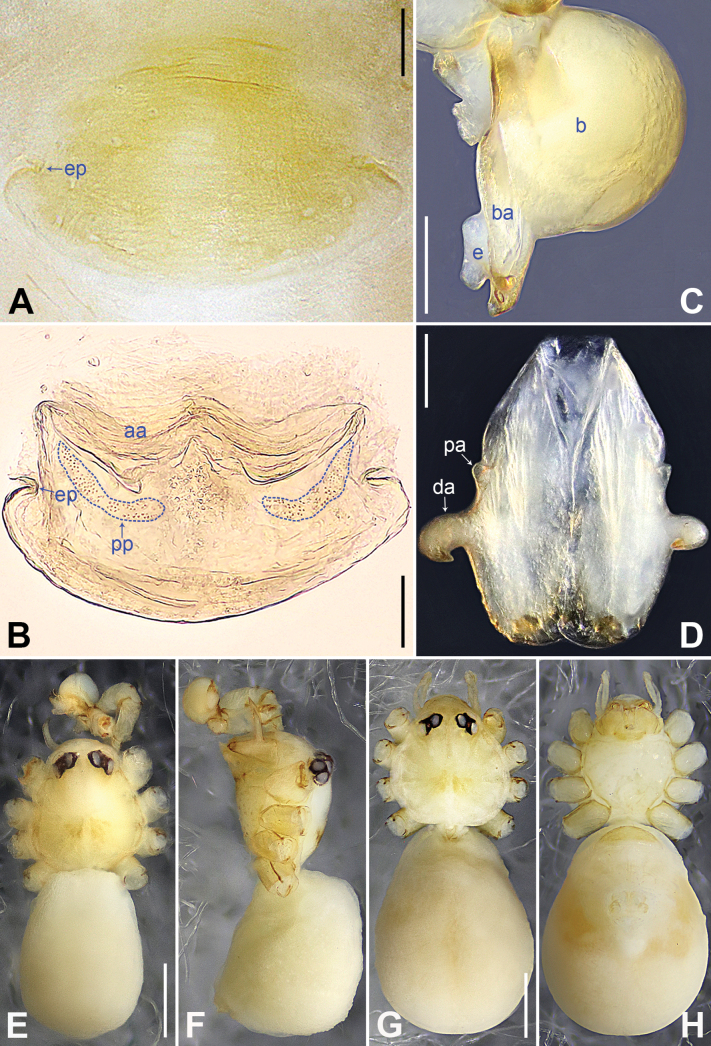
*Belisanayongcong* sp. nov., holotype male (**C–F**) and paratype female (**A, B, G, H**) **A** epigyne, ventral view **B** vulva, dorsal view **C** bulb, prolateral view **D** chelicerae, frontal view **E–H** habitus (**E, G** dorsal view **F** lateral view **H** ventral view). Abbreviations: aa = anterior arch, b = bulb, ba = bulbal apophysis, da = distal apophysis, e = embolus, ep = epigynal pocket, pa = proximo-lateral apophysis, pp = pore plate. Scale bars: 0.10 (**A–D**); 0.40 (**E–H**).

The new species resembles *B.yongcong* sp. nov. (Figs 14, 15, 23A, B, 25C, D) by having similar male chelicerae (tips of distal apophyses widely separated and pointing inwards; Fig. 13D), bulbal apophysis (hooked; Fig. 13C), and epigyne (epigynal pockets on lateral part of epigynal plate, epigynal plate posteriorly curved; Figs 13A, 25A), but can be distinguished by procursus with distal spine (arrow 1 in Figs 12C, 22K vs absent) and without ventro-subdistal membranous lamella, prolatero-distal membranous lamella, and sclerotized dorso-subdistal apophysis (Figs 12C, 22K vs present), and by vulval pore plates long elliptic (3× longer than wide, pp in Figs 13B, 25B vs curved and 9×).

#### Description.

**Male** (**holotype**): Total length 1.35 (1.48 with clypeus), prosoma 0.57 long, 0.59 wide, opisthosoma 0.78 long, 0.60 wide. Leg I: 10.06 (2.48, 0.21, 2.73, 3.36, 1.28), leg II: 6.94 (2.00, 0.22, 1.74, 2.18, 0.80), leg III: 5.08 (1.50, 0.20, 1.14, 1.64, 0.60), leg IV: 6.72 (1.94, 0.20, 1.72, 2.15, 0.71); tibia I L/d: 46. Eye interdistances and diameters: PME–PME 0.09, PME 0.05, PME–ALE 0.02. Sternum width/length: 0.45/0.40. Habitus as in Fig. 13E, F. Dorsal shield of prosoma yellowish, without marks; clypeus and sternum yellowish. Legs whitish, without darker rings. Opisthosoma yellowish, without spots. Thoracic furrow absent. Clypeus unmodified. Eye pigments indistinct. Chelicerae with pair of proximo-lateral apophyses (pa in Fig. 13D) and pair of curved distal apophyses (distance between tips: 0.30; da in Fig. 13D). Palp as in Fig. 12A, B; trochanter with ventral apophysis (as long as wide, arrow 1 in Fig. 12B); femur with retrolatero-proximal protrusion (arrow 2 in Fig. 12B); procursus simple, with distal spine (arrow 1 in Figs 12C, 22K) and retrolateral membranous flap (f in Figs 12D, 22L); bulb with hooked apophysis (ba in Fig. 13C) and simple embolus (e in Fig. 13C). Retrolateral trichobothria on tibia I at 6% proximally; legs with short vertical setae on metatarsi; tarsus I with 15 distinct pseudosegments.

**Female** (**paratype**, IZCAS-Ar45215): Similar to male, habitus as in Fig. 13G, H. Total length 1.75 (1.85 with clypeus), prosoma 0.56 long, 0.59 wide, opisthosoma 1.19 long, 0.80 wide; tibia I: 2.19; tibia I L/d: 44. Eye interdistances and diameters: PME–PME 0.09, PME 0.05, PME–ALE 0.02. Sternum width/length: 0.44/0.38. Epigyne simple and flat, posteriorly curved, with pair of lateral pockets 0.33 apart (ep in Figs 13A, 25A). Vulva with ridge-shaped anterior arch (aa in Figs 13B, 25B) and pair of long elliptic pore plates (3× longer than wide, pp in Figs 13B, 25B).

#### Variation.

Tibia I in the other two female paratypes (IZCAS-Ar45216–17): 2.10, 2.18.

#### Habitat.

The species was found in the dark zone inside the cave.

#### Distribution.

China (Guizhou, type locality; Fig. 1).

#### Etymology.

The specific name refers to the type locality; noun in apposition.

### ﻿ Belisana yongcong

Taxon classificationAnimaliaAraneaePholcidae

Wang, S. Li & Yao sp. nov.

0A3BBAAB-9E69-5516-BEF7-69327FD252EF

https://zoobank.org/1E1B4058-113E-48F6-9734-39D486FF0FB9

Figs 14 , 15 , 23A, B , 25C, D

#### Type material.

***Holotype*: China** • ♂; Guizhou, Qiandongnan, Liping County, Yongcong Town, Guantuan Village, Guantuan Cave; 25°59.028'N, 109°06.979'E; alt. 544 m; 23 May 2007; J. Liu & Y. Lin leg.; IZCAS-Ar45218. ***Paratypes*: China** • 2♂; same data as for holotype; IZCAS-Ar45219–20 • 5♀; same data as for holotype; IZCAS-Ar45221–25.

#### Diagnosis.

The new species resembles *B.nayong* sp. nov. (Figs 8, 9, 22G, H, 24G, H) by having similar bulbal apophysis (hooked; Fig. 15C) and vulva (anterior arch ridge-shaped, pore plates curved, long elliptic and 8× longer than wide; Figs 15B, 25D), but can be distinguished by procursus with blunt ventro-subdistal membranous process (arrow 1 in Figs 14C, 23A vs pointed), by male cheliceral distal apophyses pointing outwards (da in Fig. 15D vs inwards), and by epigyne with lateral pockets (ep in Figs 15A, 25C vs postero-median).

#### Description.

**Male** (**holotype**): Total length 1.73 (1.82 with clypeus), prosoma 0.83 long, 0.67 wide, opisthosoma 0.90 long, 0.66 wide. Leg I: 14.76 (3.60, 0.34, 4.05, 5.58, 1.56), leg II: 12.45 (3.36, 0.32, 3.08, 4.60, 1.09), leg III: 8.21 (2.38, 0.26, 1.98, 2.84, 0.75), leg IV: 10.64 (3.20, 0.26, 2.66, 3.72, 0.80); tibia I L/d: 46. Eye interdistances and diameters: PME–PME 0.06, PME 0.09, PME–ALE 0.02. Sternum width/length: 0.54/0.49. Habitus as in Fig. 15E, F. Dorsal shield of prosoma yellowish, with brownish radiating marks; clypeus and sternum yellowish. Legs whitish, without darker rings. Opisthosoma yellowish, without spots. Thoracic furrow absent. Clypeus unmodified. Chelicerae with pair of proximo-lateral apophyses (pa in Fig. 15D) and pair of curved distal apophyses (distance between tips: 0.32; da in Fig. 15D). Palp as in Fig. 14A, B; trochanter with ventral apophysis (as long as wide, arrow 1 in Fig. 14B); femur with retrolatero-proximal protrusion (arrow 2 in Fig. 14B); procursus simple proximally but complex distally, with ventro-subdistal membranous lamella (arrow 1 in Figs 14C, 23A) bearing proximally sclerotized part, prolatero-distal membranous lamella (arrow 2 in Figs 14C, 23A) bearing proximally sclerotized part, sclerotized dorso-subdistal apophysis (arrow 3 in Figs 14C, 23A), and retrolateral membranous flap (f in Figs 14D, 23B); bulb with hooked apophysis (ba in Fig. 15C) and simple embolus (e in Fig. 15C). Retrolateral trichobothria on tibia I at 10% proximally; legs with short vertical setae on metatarsi; tarsus I with 20 distinct pseudosegments.

**Female** (**paratype**, IZCAS-Ar45221): Similar to male, habitus as in Fig. 15G, H. Total length 2.17 (2.29 with clypeus), prosoma 0.87 long, 0.76 wide, opisthosoma 1.30 long, 1.08 wide; tibia I: 3.65; tibia I L/d: 47. Eye interdistances and diameters: PME–PME 0.09, PME 0.08, PME–ALE 0.02. Sternum width/length: 0.59/0.45. Epigyne simple and flat, posteriorly curved, with pair of lateral pockets 0.35 apart (ep in Figs 15A, 25C). Vulva with ridge-shaped anterior arch (aa in Figs 15B, 25D) and pair of curved, long elliptic pore plates (9× longer than wide, pp in Figs 15B, 25D).

#### Variation.

Tibia I in one male paratype (IZCAS-Ar45219): 5.32 (leg I missing in IZCAS-Ar45220). Tibia I in the other four female paratypes (IZCAS-Ar45222–25): 3.76, 3.85, 3.96, 4.16.

#### Habitat.

The species was found in the dark zone inside the cave.

#### Distribution.

China (Guizhou, type locality; Fig. 1).

#### Etymology.

The specific name refers to the type locality; noun in apposition.

### ﻿ Belisana zhangi

Taxon classificationAnimaliaAraneaePholcidae

Tong & Li, 2007

257E481B-38D6-5DC5-AAD1-5ABABE86F051

**Figure 16.**
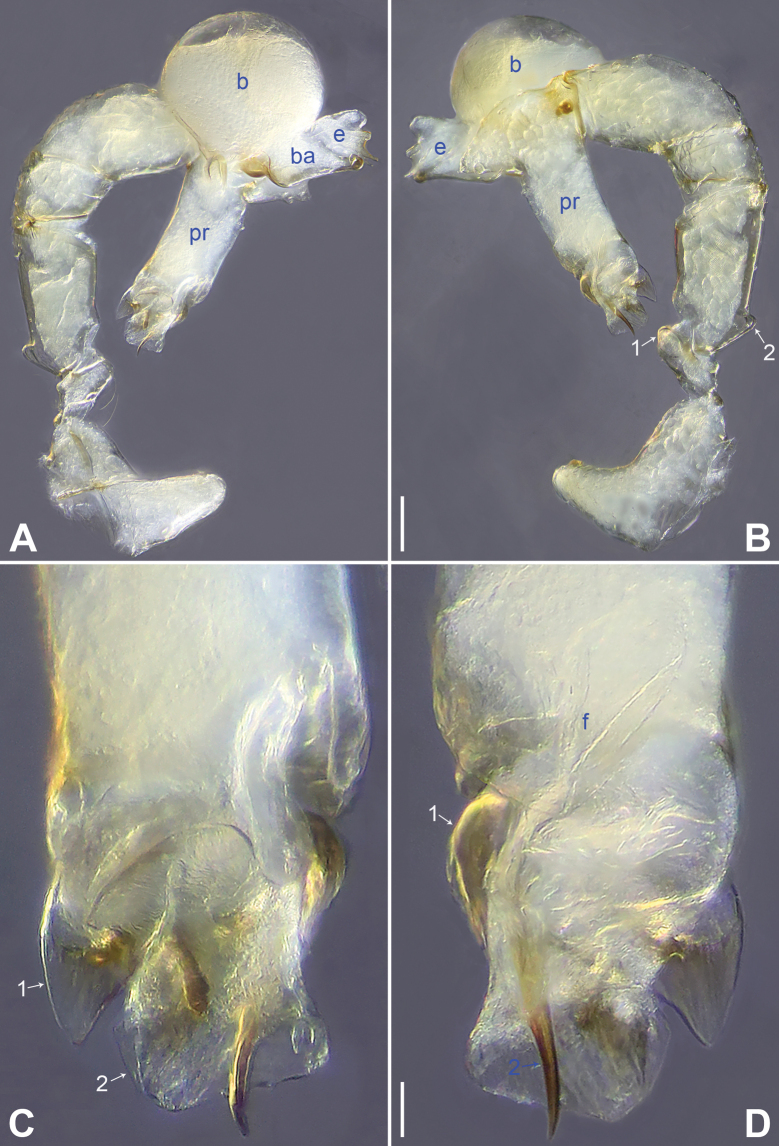
*Belisanazhangi* Tong & Li, 2007, male **A, B** palp (**A** prolateral view **B** retrolateral view, arrow 1 points at ventral apophysis, arrow 2 points at retrolatero-proximal protrusion) **C, D** distal part of procursus (**C** prolateral view, arrow 1 points at ventro-subdistal membranous lamella, arrow 2 points at prolatero-distal membranous lamella **D** retrolateral view, arrow 1 points at sclerotized dorso-subdistal apophysis, arrow 2 points at retrolatero-distal spine). Abbreviations: b = bulb, ba = bulbal apophysis, e = embolus, f = flap, pr = procursus. Scale bars: 0.10 (**A, B**); 0.02 (**C, D**).

**Figure 17.**
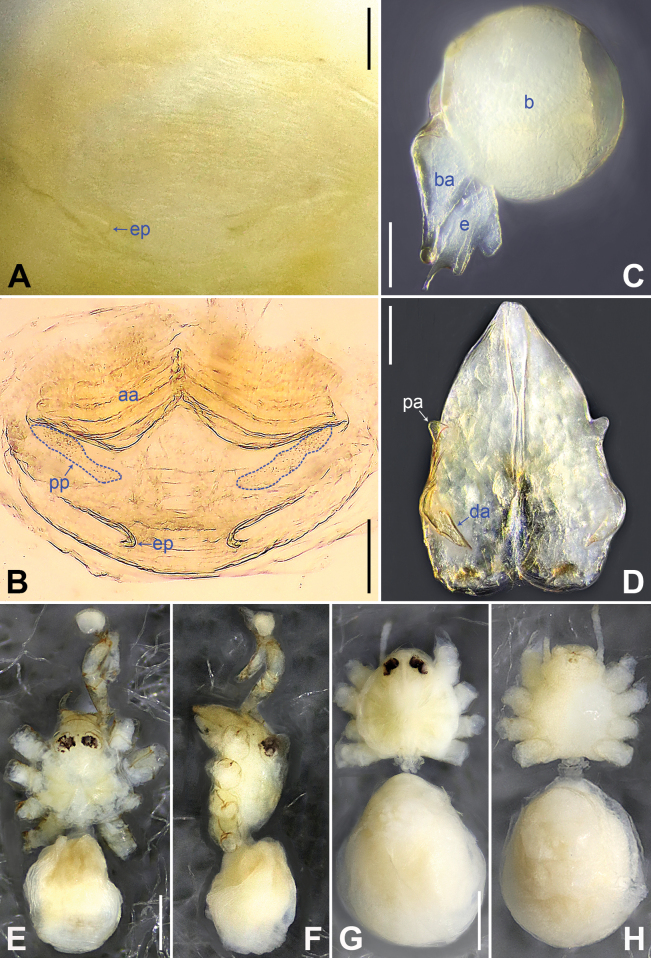
*Belisanazhangi* Tong & Li, 2007, male (**C–F**) and female (**A, B, G, H**) **A** epigyne, ventral view **B** vulva, dorsal view **C** bulb, prolateral view **D** chelicerae, frontal view **E–H** habitus (**E, G** dorsal view **F** lateral view **H** ventral view). Abbreviations: aa = anterior arch, b = bulb, ba = bulbal apophysis, da = distal apophysis, e = embolus, ep = epigynal pocket, pa = proximo-lateral apophysis, pp = pore plate. Scale bars: 0.10 (**A–D**); 0.40 (**E–H**).

Figs 16 , 17 , 23C, D , 25E, F

Belisana zhangi Tong and Li 2007: 505, figs 1–6.

#### Material examined.

**China** • 1♂; Guizhou, Anshun, Puding County, Huachu Town, Jinqian Cave; 26°14.835'N, 105°37.521'E; 3 May 2005; collector unknown; IZCAS-Ar45177 • 3♀; same data as for preceding; IZCAS-Ar45178–80.

#### Diagnosis.

The species resembles *B.yuhaoi* Yang & Yao, 2023 (Yang et al. 2023a: 178, figs 2A, B, 3A–D, 4A–H) by having similar bulbal apophysis (hooked; Fig. 17C) and epigyne (epigynal pockets on posterior part of epigynal plate, epigynal plate posteriorly curved; Figs 17A, 25E), but can be distinguished by procursus with nearly angular ventro-subdistal membranous lamella (arrow 1 in Figs 16C, 23C vs nearly round) and curved retrolateral membranous flap (f in Figs 16D, 23D vs angular), by male cheliceral distal apophyses pointing inwards (da in Fig. 17D vs downwards), and by vulval pore plates long elliptic (pp in Figs 17B, 25F vs nearly triangular).

#### Habitat.

The species was found in the dark zone inside the cave.

#### Distribution.

China (Guangxi, type locality; Guizhou, Fig. 1).

### ﻿ Belisana zhouxi

Taxon classificationAnimaliaAraneaePholcidae

Wang, S. Li & Yao sp. nov.

2266C4A0-AF5E-516F-A433-5C0A6547F024

https://zoobank.org/D2E3F0DB-7736-46DA-ADC9-9F1FC1CF3ACD

**Figure 18.**
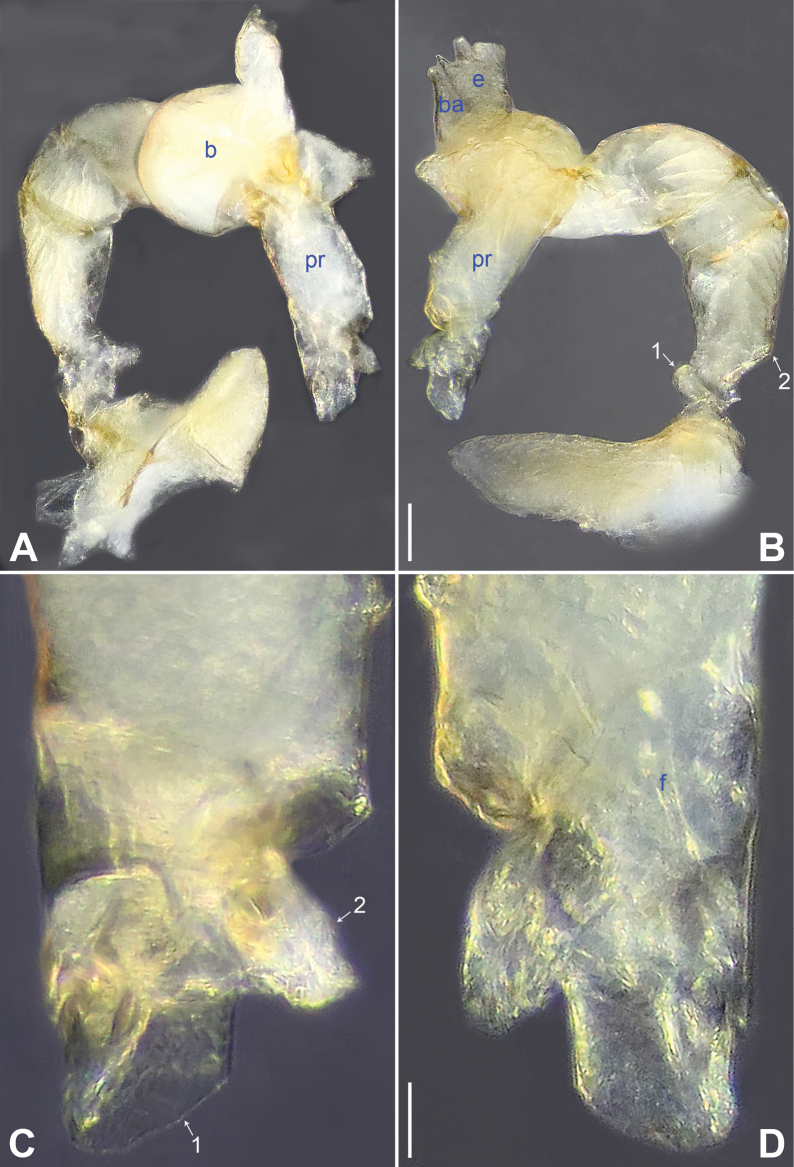
*Belisanazhouxi* sp. nov., holotype male **A, B** palp (**A** prolateral view **B** retrolateral view, arrow 1 points at ventral apophysis, arrow 2 points at retrolatero-proximal protrusion) **C, D** distal part of procursus (**C** prolateral view, arrow 1 points at prolatero-distal membranous lamella, arrow 2 points at dorso-subdistal membranous process **D** retrolateral view). Abbreviations: b = bulb, ba = bulbal apophysis, e = embolus, f = flap, pr = procursus. Scale bars: 0.05 (**A, B**); 0.02 (**C, D**).

**Figure 19.**
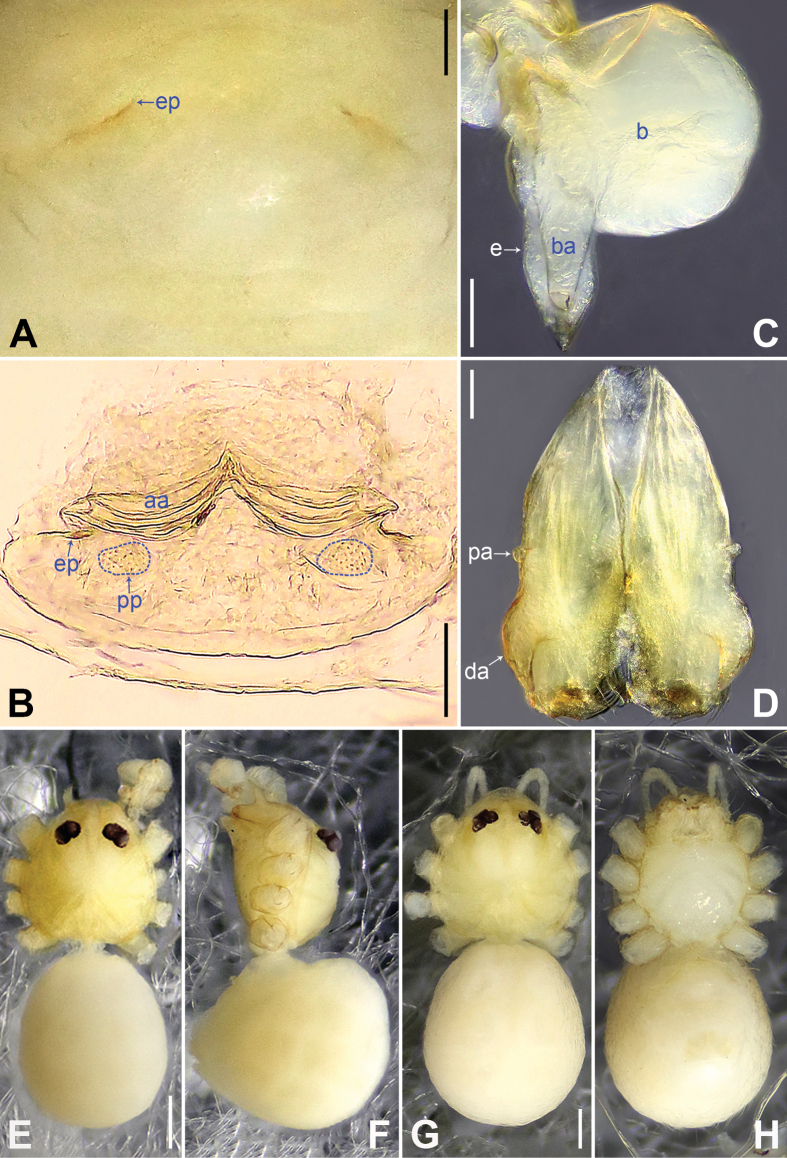
*Belisanazhouxi* sp. nov., holotype male (**C–F**) and paratype female (**A, B, G, H**) **A** epigyne, ventral view **B** vulva, dorsal view **C** bulb, prolateral view **D** chelicerae, frontal view **E–H** habitus (**E, G** dorsal view **F** lateral view **H** ventral view). Abbreviations: aa = anterior arch, b = bulb, ba = bulbal apophysis, da = distal apophysis, e = embolus, ep = epigynal pocket, pa = proximo-lateral apophysis, pp = pore plate. Scale bars: 0.05 (**A–D**); 0.20 (**E–H**).

Figs 18 , 19 , 23E, F , 25G, H

#### Type material.

***Holotype*: China** • ♂; Guizhou, Kaili, Zhouxi Town, Hebian Cave; 26°29.280'N, 107°55.442'E; alt. 665 m; 25 May 2007; J. Liu & Y. Lin leg.; IZCAS-Ar45226. ***Paratypes*: China** • 2♀; same data as for holotype; IZCAS-Ar45227–28.

#### Diagnosis.

The new species resembles *B.majiang* sp. nov. (Figs 6, 7, 22E, F, 24E, F) by having similar male chelicerae (tips of distal apophyses pointing downwards; Fig. 19D), bulbal apophysis (hooked; Fig. 19C), and epigyne (epigynal pockets on antero-lateral part of epigynal plate, epigynal plate posteriorly curved; Figs 19A, 25G), but can be distinguished by procursus without ventro-subdistal membranous process and dorso-distal spine (Figs 18C, 23E vs present) and by vulval pore plates elliptic (2× longer than wide, pp in Figs 19B, 25H vs 3×).

#### Description.

**Male** (**holotype**): Total length 1.17 (1.27 with clypeus), prosoma 0.46 long, 0.50 wide, opisthosoma 0.71 long, 0.61 wide. Leg I: 6.72 (1.84, 0.21, 1.66, 2.13, 0.88), leg II: 4.56 (1.36, 0.19, 1.06, 1.36, 0.59), leg III: 3.29 (0.96, 0.19, 0.66, 1.01, 0.47), leg IV: 4.69 (1.44, 0.18, 1.16, 1.42, 0.49); tibia I L/d: 27. Eye interdistances and diameters: PME–PME 0.08, PME 0.06, PME–ALE 0.02. Sternum width/length: 0.41/0.33. Habitus as in Fig. 19E, F. Dorsal shield of prosoma yellowish, without marks; clypeus and sternum yellowish. Legs whitish, without darker rings. Opisthosoma yellowish, without spots. Thoracic furrow absent. Clypeus unmodified. Chelicerae with pair of proximo-lateral apophyses (pa in Fig. 19D) and pair of curved distal apophyses (distance between tips: 0.19; da in Fig. 19D). Palp as in Fig. 18A, B; trochanter with ventral apophysis (as long as wide, arrow 1 in Fig. 18B); femur with retrolatero-proximal protrusion (arrow 2 in Fig. 18B); procursus simple proximally but complex distally, with prolatero-distal membranous lamella (arrow 1 in Figs 18C, 23E) bearing proximally slightly sclerotized part, dorso-subdistal membranous process (arrow 2 in Figs 18C, 23E), and retrolateral membranous flap (f in Figs 18D, 23F); bulb with hooked apophysis (ba in Fig. 19C) and simple embolus (e in Fig. 19C). Retrolateral trichobothria on tibia I at 7% proximally; legs with short vertical setae on metatarsi; tarsus I with 14 distinct pseudosegments.

**Female** (**paratype**, IZCAS-Ar45227): Similar to male, habitus as in Fig. 19G, H. Total length 1.21 (1.31 with clypeus), prosoma 0.49 long, 0.57 wide, opisthosoma 0.72 long, 0.63 wide; tibia I: 1.22; tibia I L/d: 22. Eye interdistances and diameters: PME–PME 0.07, PME 0.06, PME–ALE 0.01. Sternum width/length: 0.43/0.32. Epigyne simple and flat, posteriorly curved, with pair of antero-lateral pockets 0.16 apart (ep in Figs 19A, 25G). Vulva with ridge-shaped anterior arch (aa in Figs 19B, 25H) and pair of elliptic pore plates (2× longer than wide, pp in Figs 19B, 25H).

#### Variation.

Unknown. Leg I missing in another female paratype (IZCAS-Ar45228).

#### Habitat.

The species was found in the dark zone inside the cave.

#### Distribution.

China (Guizhou, type locality; Fig. 1).

#### Etymology.

The specific name refers to the type locality; noun in apposition.

### ﻿ Belisana zunyi

Taxon classificationAnimaliaAraneaePholcidae

Wang, S. Li & Yao sp. nov.

2EC26726-DC0E-5E33-A3A6-A4ED91089BAC

https://zoobank.org/7A299DDB-C11D-4D42-8354-65D16BC50C90

**Figure 20.**
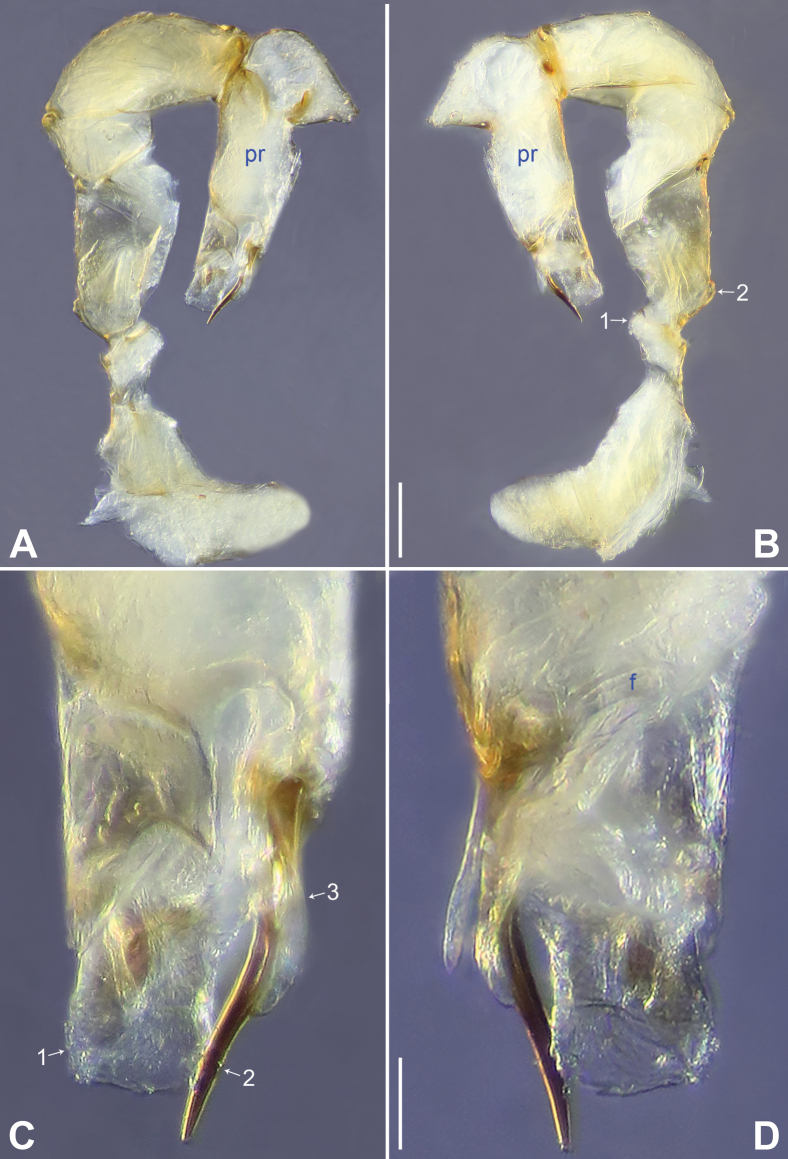
*Belisanazunyi* sp. nov., holotype male **A, B** palp (**A** prolateral view **B** retrolateral view, arrow 1 points at ventral apophysis, arrow 2 points at retrolatero-proximal protrusion) **C, D** distal part of procursus (**C** prolateral view, arrow 1 points at prolatero-distal membranous lamella, arrow 2 points at dorso-distal spine, arrow 3 points at dorso-subdistal membranous process **D** retrolateral view). Abbreviations: f = flap, pr = procursus. Scale bars: 0.10 (**A, B**); 0.03 (**C, D**).

**Figure 21.**
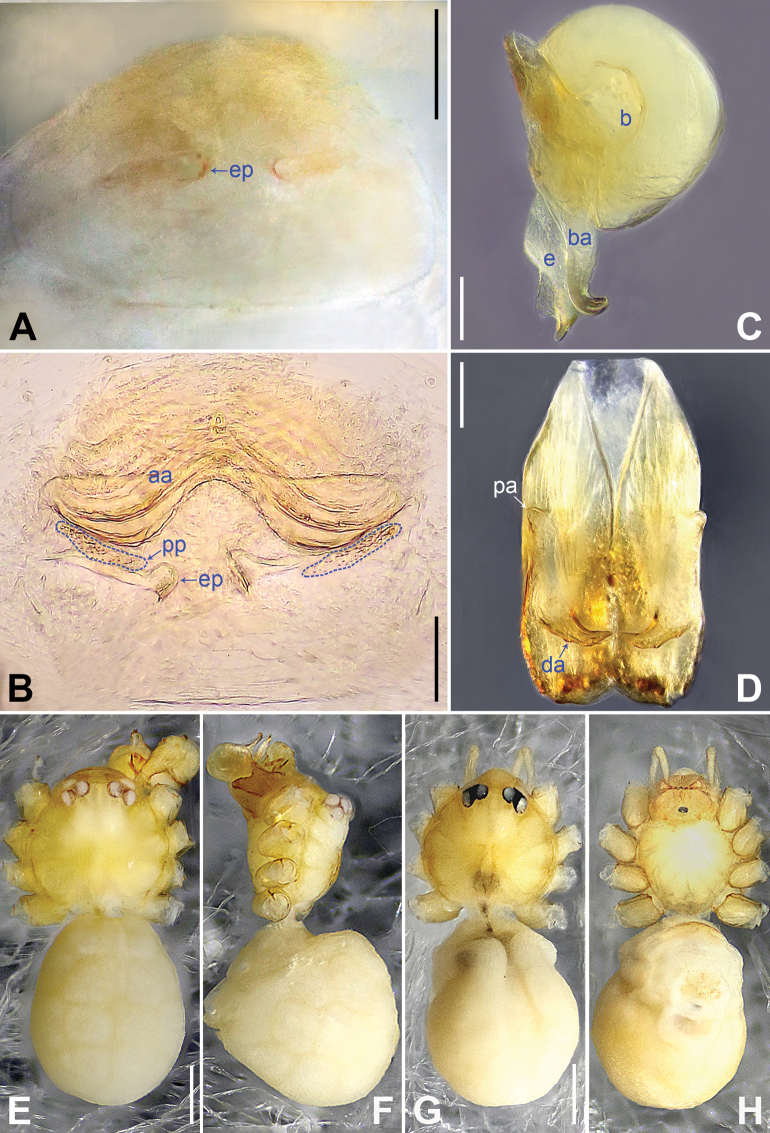
*Belisanazunyi* sp. nov., holotype male (**C–F**) and paratype female (**A, B, G, H**) **A** epigyne, ventral view **B** vulva, dorsal view **C** bulb, prolateral view **D** chelicerae, frontal view **E–H** habitus (**E, G** dorsal view **F** lateral view **H** ventral view). Abbreviations: aa = anterior arch, b = bulb, ba = bulbal apophysis, da = distal apophysis, e = embolus, ep = epigynal pocket, pa = proximo-lateral apophysis, pp = pore plate. Scale bars: 0.10 (**A–D**); 0.30 (**E–H**).

Figs 20 , 21 , 23G, H , 25I, J

#### Type material.

***Holotype*: China** • ♂; Guizhou, Zunyi, Tongzi County, Leishanguan Town, Yangliuping Village; 28°08.692'N, 106°47.378'E; alt. 1008 m; 11 May 2007; J. Liu & Y. Lin leg.; IZCAS-Ar45229. ***Paratypes*: China** • 4♂; same data as for holotype; IZCAS-Ar45230–33 • 13♀; same data as for holotype; IZCAS-Ar45234–46.

#### Diagnosis.

The new species resembles *B.zhangi* Tong & Li, 2007 (Tong and Li 2007: 505, figs 1–6) by having similar bulbal apophysis (hooked; Fig. 21C) and vulva (anterior arch ridge-shaped, pore plates long elliptic and 6× longer than wide; Figs 21B, 25J), but can be distinguished by procursus with dorso-subdistal membranous process (arrow 3 in Figs 20C, 23G vs sclerotized apophysis) and without ventro-subdistal membranous lamella (Figs 20C, 23G vs present), by tips of male cheliceral distal apophyses close to each other (da in Fig. 21D vs widely separated), and by epigyne with median pockets (ep in Figs 21A, 25I vs postero-median).

**Figure 22.**
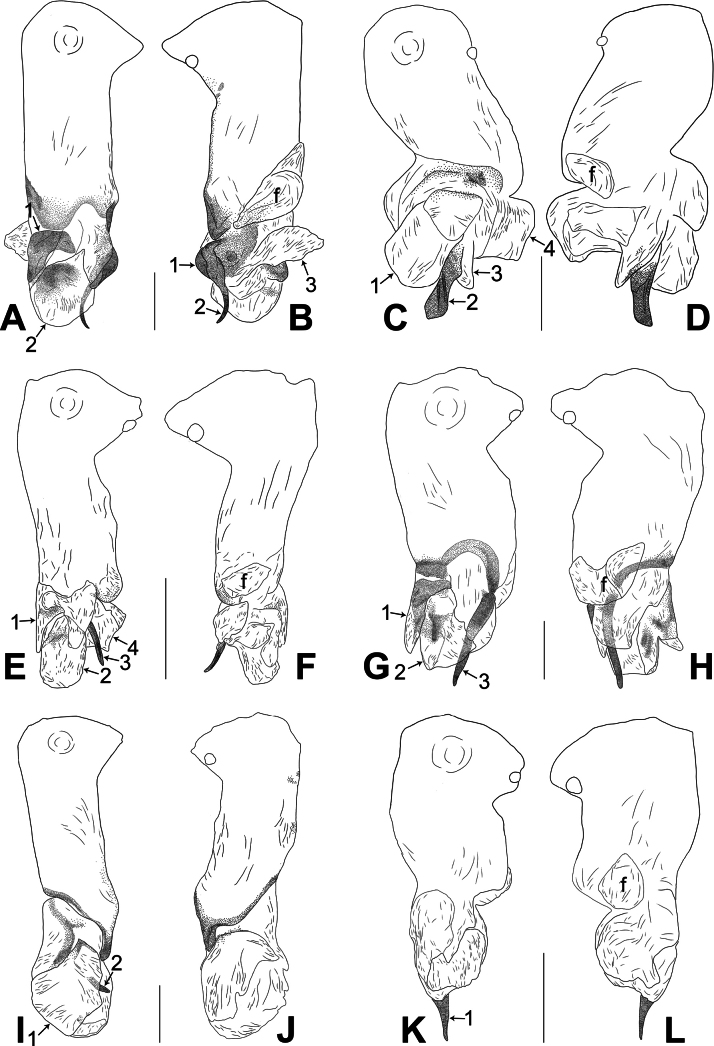
Procursus in prolateral and retrolateral views (The arrows point at the same structures as those shown in the photos of each species) **A, B***Belisanabijie* sp. nov. **C, D***B.liupanshui* sp. nov. **E, F***B.majiang* sp. nov. **G, H***B.nayong* sp. nov. **I, J***B.qixingguan* sp. nov. **K, L***B.xiuwen* sp. nov. Abbreviation: f = flap. Scale bars: 0.10.

**Figure 23.**
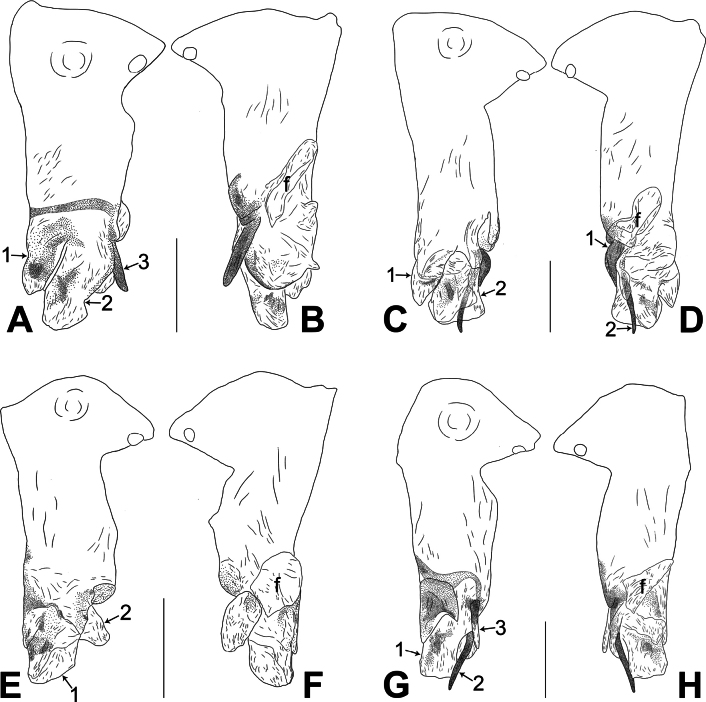
Procursus in prolateral and retrolateral views (The arrows point at the same structures as those shown in the photos of each species) **A, B***Belisanayongcong* sp. nov. **C, D***B.zhangi* Tong & Li, 2007 **E, F***B.zhouxi* sp. nov. **G, H***B.zunyi* sp. nov. Abbreviation: f = flap. Scale bars: 0.10.

**Figure 24.**
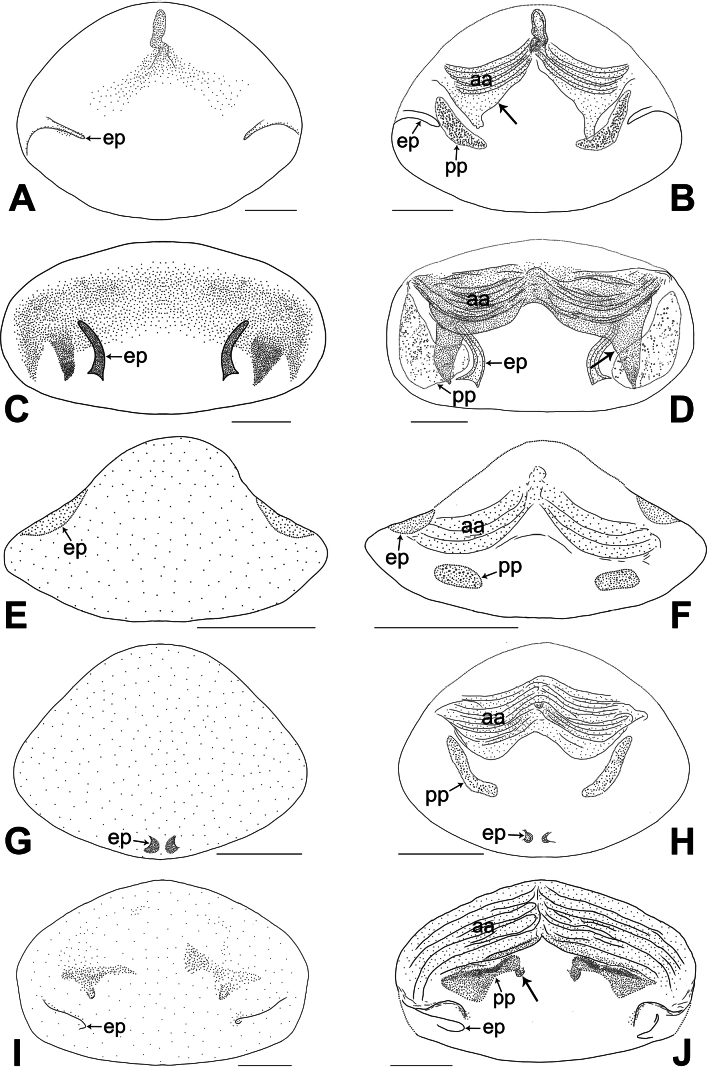
Female genitalia in ventral and dorsal views **A, B***Belisanabijie* sp. nov., arrow points at lateral sclerite **C, D***B.liupanshui* sp. nov., arrow points at lateral sclerite **E, F***B.majiang* sp. nov. **G, H***B.nayong* sp. nov **I, J***B.qixingguan* sp. nov., arrow points at sclerotized protrusion. Abbreviations: aa = anterior arch, ep = epigynal pocket, pp = pore plate. Scale bars: 0.10.

**Figure 25.**
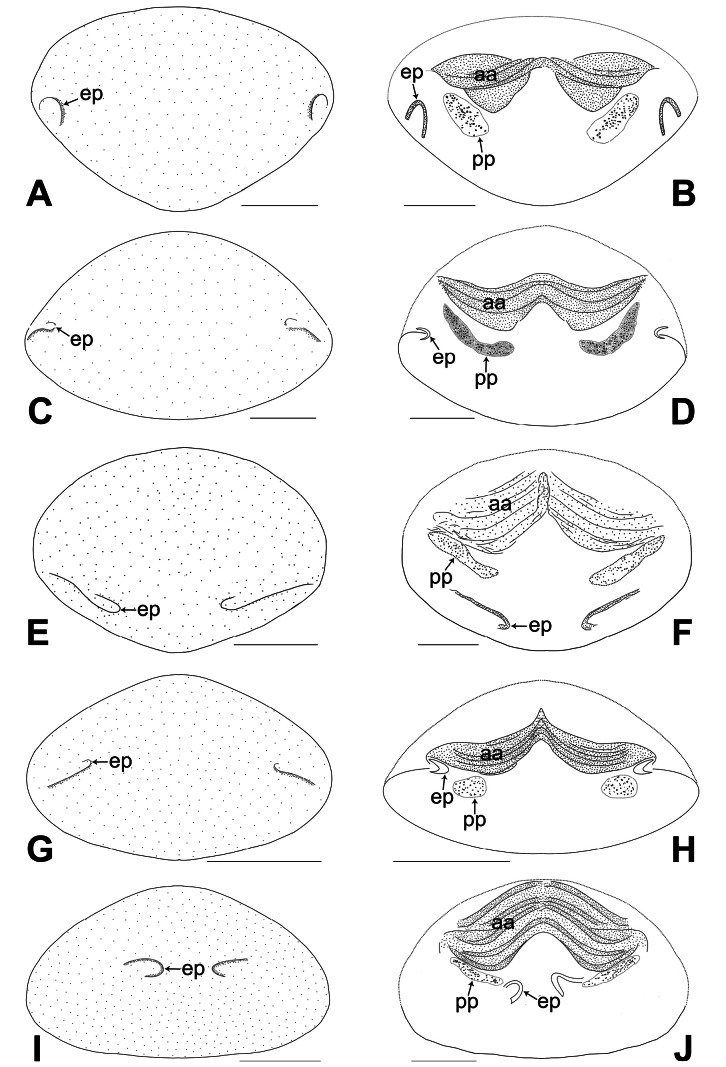
Female genitalia in ventral and dorsal views **A, B***Belisanaxiuwen* sp. nov. **C, D***B.yongcong* sp. nov. **E, F***B.zhangi* Tong & Li, 2007 **G, H***B.zhouxi* sp. nov. **I, J***B.zunyi* sp. nov. Abbreviations: aa = anterior arch, ep = epigynal pocket, pp = pore plate. Scale bars: 0.10.

#### Description.

**Male** (**holotype**): Total length 1.62 (1.72 with clypeus), prosoma 0.60 long, 0.66 wide, opisthosoma 1.02 long, 0.78 wide. Leg I: 13.53 (3.60, 0.29, 3.50, 4.60, 1.54), leg II: 8.40 (2.25, 0.30, 2.08, 2.81, 0.96), leg III: 5.69 (1.64, 0.25, 1.34, 1.86, 0.60), leg IV: 7.72 (2.12, 0.24, 2.00, 2.56, 0.80); tibia I L/d: 43. Eye interdistances and diameters: PME–PME 0.09, PME 0.08, PME–ALE 0.02. Sternum width/length: 0.53/0.47. Habitus as in Fig. 21E, F. Dorsal shield of prosoma yellowish, without marks; clypeus and sternum yellowish. Legs whitish, without darker rings. Opisthosoma yellowish, without spots. Thoracic furrow absent. Clypeus unmodified. Chelicerae with pair of proximo-lateral apophyses (pa in Fig. 21D) and pair of curved distal apophyses (distance between tips: 0.04; da in Fig. 21D). Palp as in Fig. 20A, B; trochanter with ventral apophysis (as long as wide, arrow 1 in Fig. 20B); femur with retrolatero-proximal protrusion (arrow 2 in Fig. 20B); procursus simple proximally but complex distally, with prolatero-distal membranous lamella (arrow 1 in Figs 20C, 23G) bearing proximally sclerotized part, dorso-distal spine (arrow 2 in Figs 20C, 23G), dorso-subdistal membranous process (arrow 3 in Figs 20C, 23G), and retrolateral membranous flap (f in Figs 20D, 23H); bulb with hooked apophysis (ba in Fig. 21C) and simple embolus (e in Fig. 21C). Retrolateral trichobothria on tibia I at 10% proximally; legs with short vertical setae on metatarsi; tarsus I with 15 distinct pseudosegments.

**Female** (**paratype**, IZCAS-Ar45234): Similar to male, habitus as in Fig. 21G, H. Total length 1.57 (1.66 with clypeus), prosoma 0.60 long, 0.68 wide, opisthosoma 0.97 long, 0.78 wide; tibia I: 2.33; tibia I L/d: 35. Eye interdistances and diameters: PME–PME 0.08, PME 0.06, PME–ALE 0.02. Sternum width/length: 0.50/0.46. Epigyne simple and flat, posteriorly straight, with pair of median pockets 0.06 apart (ep in Figs 21A, 25I). Vulva with ridge-shaped anterior arch (aa in Figs 21B, 25J) and pair of long elliptic pore plates (6× longer than wide, pp in Figs 21B, 25J).

#### Variation.

Tibia I in four male paratypes (IZCAS-Ar45230–45233): 3.12, 3.20, 3.28, 3.70. Tibia I in the other 12 female paratypes (IZCAS-Ar45235–46): 2.31–2.50.

#### Habitat.

The species was found in the dark zone inside the cave.

#### Distribution.

China (Guizhou, type locality; Fig. 1).

#### Etymology.

The specific name refers to the type locality; noun in apposition.

## ﻿Discussion

*Belisana* is highly diverse in southern China. Including the nine species described in this paper, there are now 83 species of *Belisana* in southern China, representing 49% of the global total of the genus. Thailand, Indonesia, and Vietnam rank second, third, and fourth, respectively, in species diversity of *Belisana*; however, these countries have recorded only 19, 18, and 17 species, respectively. Other countries, such as Laos (8 spp.), Malaysia (8 spp.), and Sri Lanka (6 spp.), have recorded fewer than ten species (WSC 2024). This high level of activity in China contrasts with the sporadic coverage of Southeast Asia, where most research has been conducted by foreign arachnologists, and several countries lack native expertise in this field (Yao and Li 2021; Zhang et al. 2022; Yang et al. 2023b). Given that Southeast Asia encompasses the Indo-Burma and Sundaland biodiversity hotspots, we anticipate that further exploration will reveal additional, as-yet-undiscovered species diversity of *Belisana*.

## Supplementary Material

XML Treatment for Belisana

XML Treatment for Belisana bijie

XML Treatment for Belisana liupanshui

XML Treatment for Belisana majiang

XML Treatment for Belisana nayong

XML Treatment for Belisana qixingguan

XML Treatment for Belisana xiuwen

XML Treatment for Belisana yongcong

XML Treatment for Belisana zhangi

XML Treatment for Belisana zhouxi

XML Treatment for Belisana zunyi

## ﻿Additional information

### Author ORCIDs

Bing Wang https://orcid.org/0009-0009-7047-4395

Jinglin Li https://orcid.org/0009-0008-3932-309X

Shuqiang Li https://orcid.org/0000-0002-3290-5416

Zhiyuan Yao https://orcid.org/0000-0002-1631-0949

### Data availability

All of the data that support the findings of this study are available in the main text.
